# The *Clostridium* small RNome that responds to stress: the paradigm and importance of toxic metabolite stress in *C. acetobutylicum*

**DOI:** 10.1186/1471-2164-14-849

**Published:** 2013-12-04

**Authors:** Keerthi P Venkataramanan, Shawn W Jones, Kevin P McCormick, Sridhara G Kunjeti, Matthew T Ralston, Blake C Meyers, Eleftherios T Papoutsakis

**Affiliations:** 1 Department of Chemical and Biomolecular Engineering, University of Delaware, Newark, DE, USA; 2 Delaware Biotechnology Institute, University of Delaware, Newark, DE, USA; 3 Department of Computer and Information Sciences, University of Delaware, Newark, DE, USA; 4 Department of Plant and Soil Sciences, University of Delaware, Newark, DE, USA; 5 Center for Bioinformatics and Computational Biology, University of Delaware, Newark, DE, USA

## Abstract

**Background:**

Small non-coding RNAs (sRNA) are emerging as major components of the cell’s regulatory network, several possessing their own regulons. A few sRNAs have been reported as being involved in general or toxic-metabolite stress, mostly in Gram^-^ prokaryotes, but hardly any in Gram^+^ prokaryotes. Significantly, the role of sRNAs in the stress response remains poorly understood at the genome-scale level. It was previously shown that toxic-metabolite stress is one of the most comprehensive and encompassing stress responses in the cell, engaging both the general stress (or heat-shock protein, HSP) response as well as specialized metabolic programs.

**Results:**

Using RNA deep sequencing (RNA-seq) we examined the sRNome of *C. acetobutylicum* in response to the native but toxic metabolites, butanol and butyrate. 7.5% of the RNA-seq reads mapped to genome outside annotated ORFs, thus demonstrating the richness and importance of the small RNome. We used comparative expression analysis of 113 sRNAs we had previously computationally predicted, and of annotated mRNAs to set metrics for reliably identifying sRNAs from RNA-seq data, thus discovering 46 additional sRNAs. Under metabolite stress, these 159 sRNAs displayed distinct expression patterns, a select number of which was verified by Northern analysis. We identified stress-related expression of sRNAs affecting transcriptional (6S, S-box & *solB*) and translational (*tmRNA & SRP-RNA*) processes, and 65 likely targets of the RNA chaperone Hfq.

**Conclusions:**

Our results support an important role for sRNAs for understanding the complexity of the regulatory network that underlies the stress response in *Clostridium* organisms, whether related to normophysiology, pathogenesis or biotechnological applications.

## Background

Small non-coding regulatory-RNAs (sRNAs), discovered on the genome of all bacteria so far examined, have been established as an integral component of the regulatory system of the cell [1-3]. Unlike their counterparts in eukaryotes, which are about 20 nucleotides long, sRNAs in bacteria span a wider size range between 50 to 500 nts [4]. Regulation of gene expression at post-transcriptional level by sRNAs has been established in both Gram^-^, such as *Vibrio fischeri*[5], *Pseudomonas aeruginosa*[6], and *Escherichia coli*[1,7], and Gram^+^ bacteria, such as *Bacillus subtilis*[8], *Listeria monocytogenes*[9] and *Streptococcus pyogenes*[10]. Identification of sRNAs in bacteria has been carried out experimentally using whole genome microarrays, intergenomic tiling arrays, shotgun cloning and, recently, RNA deep sequencing (RNA-seq) [7,11-14]. *In silico* prediction of sRNAs has been carried out using comparative genomic analyses by employing algorithms such as SIPHT [15], QRNA [16], ISI [17], and sRNAscanner [18]. Experimental detection of sRNAs that are expressed only under specific culture conditions may not be successful at other conditions, while computational methods relying on sequence conservation may not identify species-specific sRNAs. Hence, a combination of the two approaches should be logically preferable.

A number of sRNAs have been identified to play an important role in the response to stress in *Escherichia coli*, such as in oxidative stress (*OxyS*) [19], cold shock (*SraF, SraG* and *SraJ*) [1], iron depletion (*RyhB*) [20-22] and sugar stress (*SgrS*) [23]. The best and most celebrated case so far uncovered is the regulation of the major stress sigma factor, RpoS, in *E. coli*. RpoS orchestrates the cellular response to a variety of stresses and the transition to the stationary phase, and is regulated at the post-transcriptional level by several sRNAs. DsrA, RprA and ArcZ are positive regulators the RpoS expression, while OxyS is a negative regulator [24-26]. Yet, little is known regarding a role of sRNAs in the stress response of Gram^+^ prokaryotes, and nothing about the role of sRNAs in the response to chemical stress. Here we are focusing on the stress-responsive small RNome of *Clostridium acetobutylicum*, a model organism for the *Clostridium* genus and more broadly the anaerobic endospore formers [27]. *Clostridium* organisms are Gram^+^, endospore-forming firmicutes capable of fermenting a very broad set of substrates and are of great importance in human and animal pathogenesis and health, cellulose degradation, non-photosynthetic CO_2_ fixation, bioremediation and biotechnology, such as for the production of solvents and other chemicals in the context of biofuel and biorefinery applications [27,28].

The response to chemical stress, whether from autologous metabolites or allogeneic toxic chemicals (such as from carboxylic acids, high H^+^ concentrations (low pH), antibiotics, and solvents like ethanol and butanol), plays a major role in cell physiology. Chemical stress affects cell survival, metabolism, sporulation and pathogenesis in physiological milieus, such as the gut microbiome [29], and pathogenesis [19,30-32], and the natural environment. Chemical stress is a major and well recognized problem in modern bioprocessing due to toxic substrates and desirable or undesirable toxic metabolites [33]. Chemical stress in *Clostridium* organisms engages the general stress response, better known as the heat-shock protein (HSP) response, as well as more specialized responses. The HSP response involves strong upregulation of all major HSP proteins, including those of the GroESL and DnaKJ systems. Specialized responses include the acid resistance systems under acid stress [34-36], and changes in metabolic and biosynthetic programs in response to both acid and solvent stress [35,37-39]. Thus, chemical stress is one of the broadest stress responses known in this and other prokaryotes, and as such, understanding the stress-related small RNome under chemical stress is of broad and general interest.

*C. acetobutylicum* carries out the biphasic ABE (acetone-butanol-ethanol) fermentation, which consists of an acidogenic exponential phase resulting in the production of butyrate and acetate, followed by the solventogenic stationary phase characterized by the production of acetone, butanol and ethanol, and driven by the reassimilation of the acids. Using a SIPHT-based comparative genomics method, we recently predicted the existence of 113 sRNAs in *C. acetobutylicum*, among which 31 were validated by either Q-RT-PCR or Northern analysis [40]. The goal of this study is to identify sRNAs, at the genome scale, that respond to butanol and/or butyrate stress and possibly start assigning mechanistic roles for these sRNAs. sRNAs that modulate the stress response can be engaged to engineer strains tolerant to these toxic metabolites, as we and others have recently reported for both *C. acetobutylicum*[34] and *Escherichia coli*[36,41].

## Results and discussion

### A large set of temporal RNA-seq data is essential for discovery

Using RNA-seq, we aimed to identify sRNAs (previously predicted [40] and novel) that are differentially expressed under butanol and butyrate stress. To do so, we aimed to collect a large set of temporal data, which, based on our experience are more likely to lead to robust discovery outcomes [35,38,39,42]. Cultures of *C. acetobutylicum* were grown in batch mode in 4-L bioreactors up to the mid-exponential phase of growth (O.D ~ 1.0), at which point the cultures were stressed with three different concentrations of butanol and butyric acid, respectively, in 3 biological-replicate experiments each. For butanol stress experiments, the cultures were stressed with 30 mM (low), 60 mM (medium) and 90 mM (high), while for butyric-acid stress, 30 mM (low), 40 mM (medium) and 50 mM (high) butyrate concentrations were used. These levels of metabolite stress were chosen based on prior studies [35,38,39] and preliminary experiments to achieve the desirable low, medium or strong metabolic response to the applied stress. Cultures were sampled at 15, 30, 60 and 75 min post stress for RNA isolation and sequencing. These sampling times, which are of the order of the doubling time of these cells, were meant to capture largely the direct and immediate impact of these stresses on gene expression and the small RNome. Following RNA isolation, mRNA and sRNA enrichment, cDNA generation, adapter ligations and indexing, libraries were deep sequenced using Illumina’s second generation HiSeq 2000 with a read length of 50 bp.

High sequencing depth was observed for all 84 sequenced libraries from samples representing 7 distinct culture conditions with 4 time points and 3 biological replicates each. On average, for each sequenced library, 18,162,979 total reads were obtained, which are indicative of a high sequencing depth (Additional file 1). From these, for each sequenced library, 9,537,317 reads were mapped into the genome with 884,618 distinct reads after discarding unreliable reads. 46.5% of the reads mapped to annotated genes, while 2% of the reads were mapped to the 113 sRNA we have previously predicted [40]. The balance reads were mapped to structural RNA components (47%) and interoperonic (IOR; genomic DNA between operons [43]) and intergenic regions (IGR; DNA between annotated ORFs) (5.5%). These data suggest that the stress transcriptome is very rich in transcripts beyond those coded by ORFs and rRNA components.

### Read count distribution and metrics for robust identification of sRNAs

**Figure 1:**
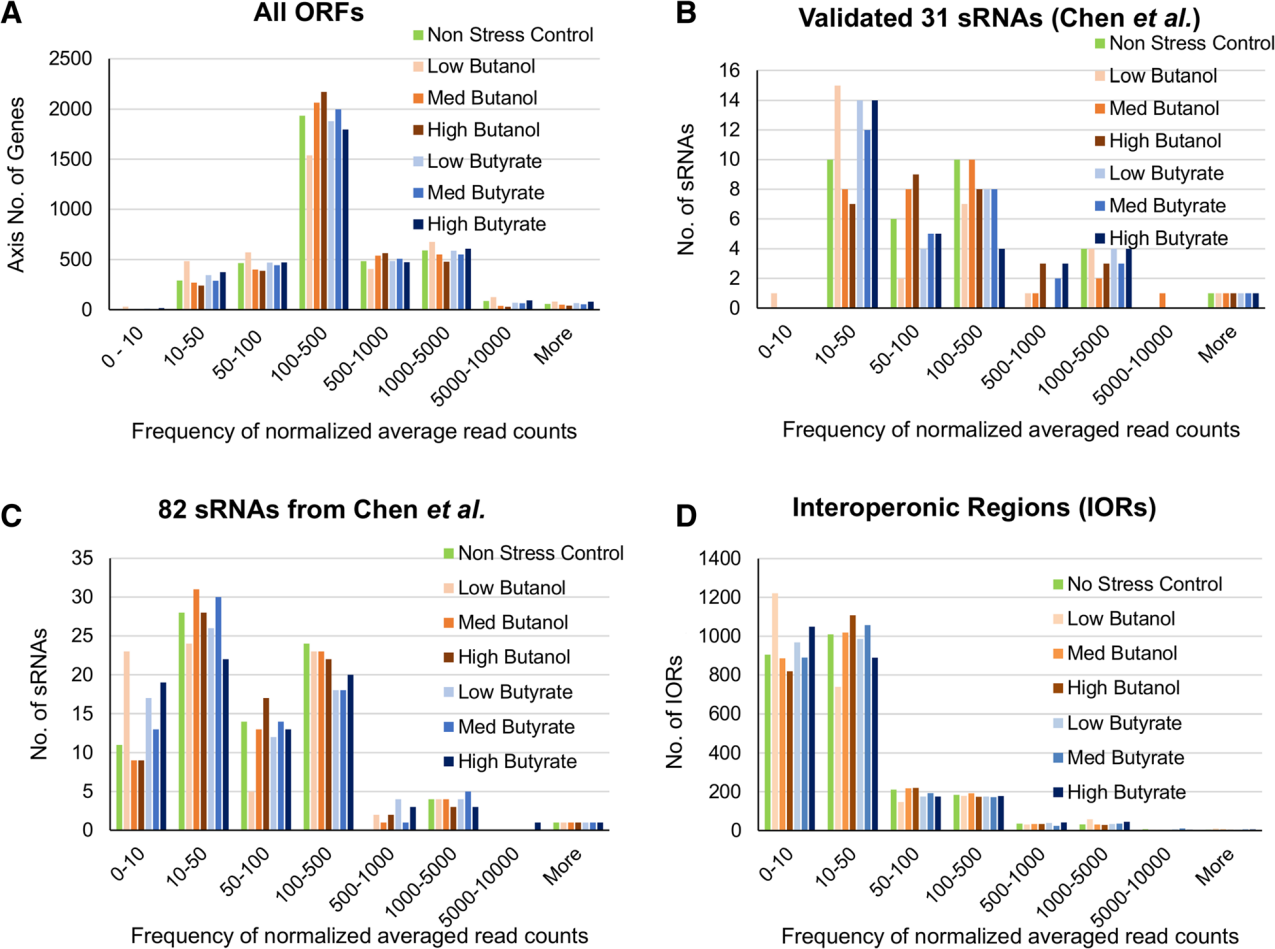
The frequency distribution of the read counts for all annotated ORFs (A); 31 of the 113 predicted sRNAs that were previously validated by Q-RT-PCR and /or Northern analysis (Chen et al. 2011) (B); the remaining 82 of the 113 predicted but not validated sRNAs (C); and all interoperonic regions (IORs) (D).

Aiming to identify novel sRNAs and also assess which sRNAs are transcribed as part of the stress transcriptome, we desired to set metrics that would allow us to call experimental reads from the RNA-seq data as factually identifying sRNAs. To do so, we used two criteria for identifying novel sRNAs on IORs. First, we selected IORs with RNA-seq expression exceeding a minimal value of read counts based on the previously annotated sRNAs as well as annotated ORFs (protein coding mRNAs) (Figure 1). The majority (ca. 75%) of annotated mRNAs had a minimum of 50 read counts (Figure 1A). The 113 sRNAs we had previously predicted [40] were divided into two categories: 31 previously validated sRNAs and the balance of 82 sRNAs (Figure 1B and 1C). As expected, read counts for sRNAs were lower than read counts for mRNAs. The majority of the previously validated 31 sRNAs had read counts over 50 (Figure 1B). The remaining 82 sRNAs had a read count distribution more skewed towards lower read counts (Figure 1C). Based on these data, we chose 50 as the read count that would most robustly identify IORs containing new sRNAs. No effort was made in this study to identify sRNAs coded on the opposite strand of annotated ORFs. Using this “minimum 50” read count criterion, 729 IORs were identified as possibly containing novel sRNAs.

**Figure 2:**
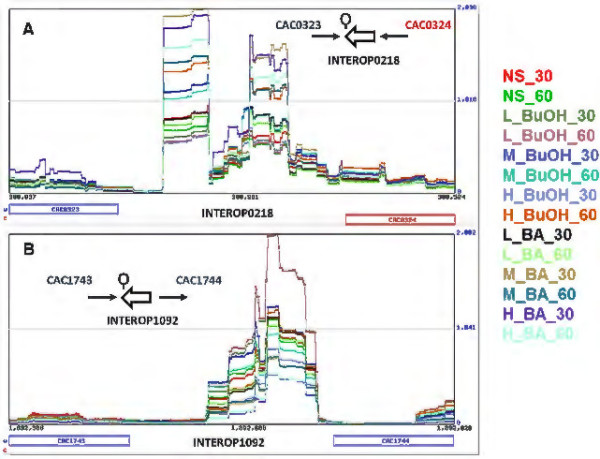
**Custom web viewer to analyze the RNA-seq data and predict novel sRNAs in *C. acetobutylicum.*** RNA-seq data screenshots of two predicted candidate sRNAs and their orientation (thick arrows), neighboring genes (→/←) and Rho-independent terminators ( Ϙ) are shown. The sRNA in **(A)** INTEROP0218 (sCAC381; a predicted Hfq target: see text) was validated by Northern analysis (Figure 5), while **(B)** sCAC1893 (INTEROP1092) was found to contain σ^G^ element (Additional File 1). BuOH- butanol, BA- butyrate, NS- no stress, 30 & 60 min post stress.

As the second selection criterion, SIPHT-based computational analysis for predicting sRNAs in the genome of *C. acetobutylicum* was performed, and 79 sRNA candidates, in addition to the previously identified 113 sRNAs, were found to be present within these 729 IORs. These were chosen for further analysis. To minimize false positives, we eliminated from the candidate list IORs having read counts predominantly from the 5’ and 3’ untranslated regions (UTRs) of the neighboring ORFs (genes), provided the neighboring ORFs also had a significant read counts (≥ 50). This elimination process was executed with the aid of a custom web viewer built to visually analyze the RNA-seq data (Figure 2). Following the screening for false positives, we successfully identified 46 novel sRNAs (Additional file 1).

### What sRNAs are expressed and differentially expressed under metabolite stress

We examined the expression profiles of the 159 (113 previously identified and the newly identified 46) sRNAs aiming to identify which are expressed and differentially expressed under the various metabolite-stress conditions. 114 of the 159 sRNAs had a minimum expression of 50 read counts in 20% of the sequenced libraries, while 70 of the 159 sRNAs had read counts over 50 read counts for 90% of the sequenced libraries representing a very broad set of culture conditions. Expression of genes and sRNAs are specific to culture conditions and not all of them are expressed at all culture conditions. Thus, expression of over 60% of the predicted sRNAs under all culture conditions in this study provides strong support for an important role of sRNAs in orchestrating the cellular response to metabolite stress.

**Figure 3:**
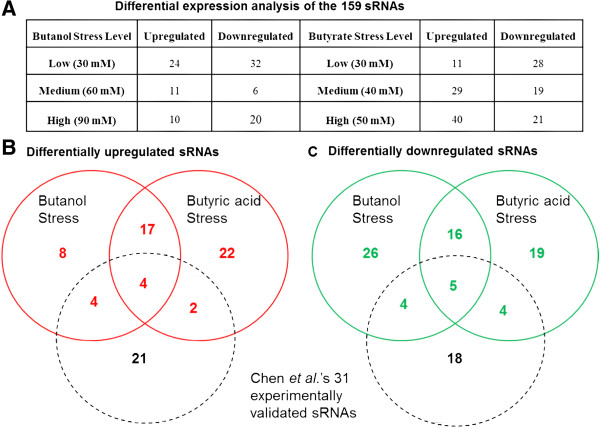
**Differential expression analysis of the 159 sRNAs under metabolite stress. (A)** Table representing the output of the differential expression analysis of the 159 sRNAs under butanol and butyrate stress. Comparison of the differentially upregulated **(B)** and downregulated **(C)** sRNAs under stress with each other. The black circle represents the comparison between the differentially expressed sRNAs under metabolite stress with the subset of 31 experimentally validated sRNAs from Chen et al. (2011).

Using pair-wise (for each time point) analysis of the 159 sRNAs for each level of metabolite stress against the unstressed control, we identified sRNAs that were differentially expressed with a p-value (DEseq analysis, Bioconductor package) ≤ 0.05. Under both butanol and butyrate stress, the number of differentially expressed sRNAs were found to be dependent on the level of stress (Figure 3A). For example, we identified 32 of the 159 sRNAs as being downregulated under low butanol stress (Figure 3A). In contrast, under medium and higher levels of butanol stress, the number of downregulated sRNAs was significantly lower. Under butyrate stress, the largest number of downregulated sRNAs was found at low levels of stress, as well. This larger number of differentially downregulated genes under lower stress levels was also observed in the mRNA expression analysis (Additional file 2: Table S1).

Butyrate stress gave rise to more (45) differentially upregulated sRNAs than butanol stress (33), while butanol stress had more differentially downregulated sRNAs (51) compared to butyrate (44). 42 sRNAs were differentially expressed under both metabolite stresses: 21 were upregulated and 21 were downregulated under both stresses (Figure 3B & 3C). Although the two metabolite stresses result in differential expression of specific sets of sRNAs that are stress and dose dependent, we found a considerable conservation of expression patterns for the two stressors among these sRNAs, thus suggesting a possible role of these sRNAs in the general stress response.

### Northern analysis of select stress-related sRNAs

**Figure 4:**
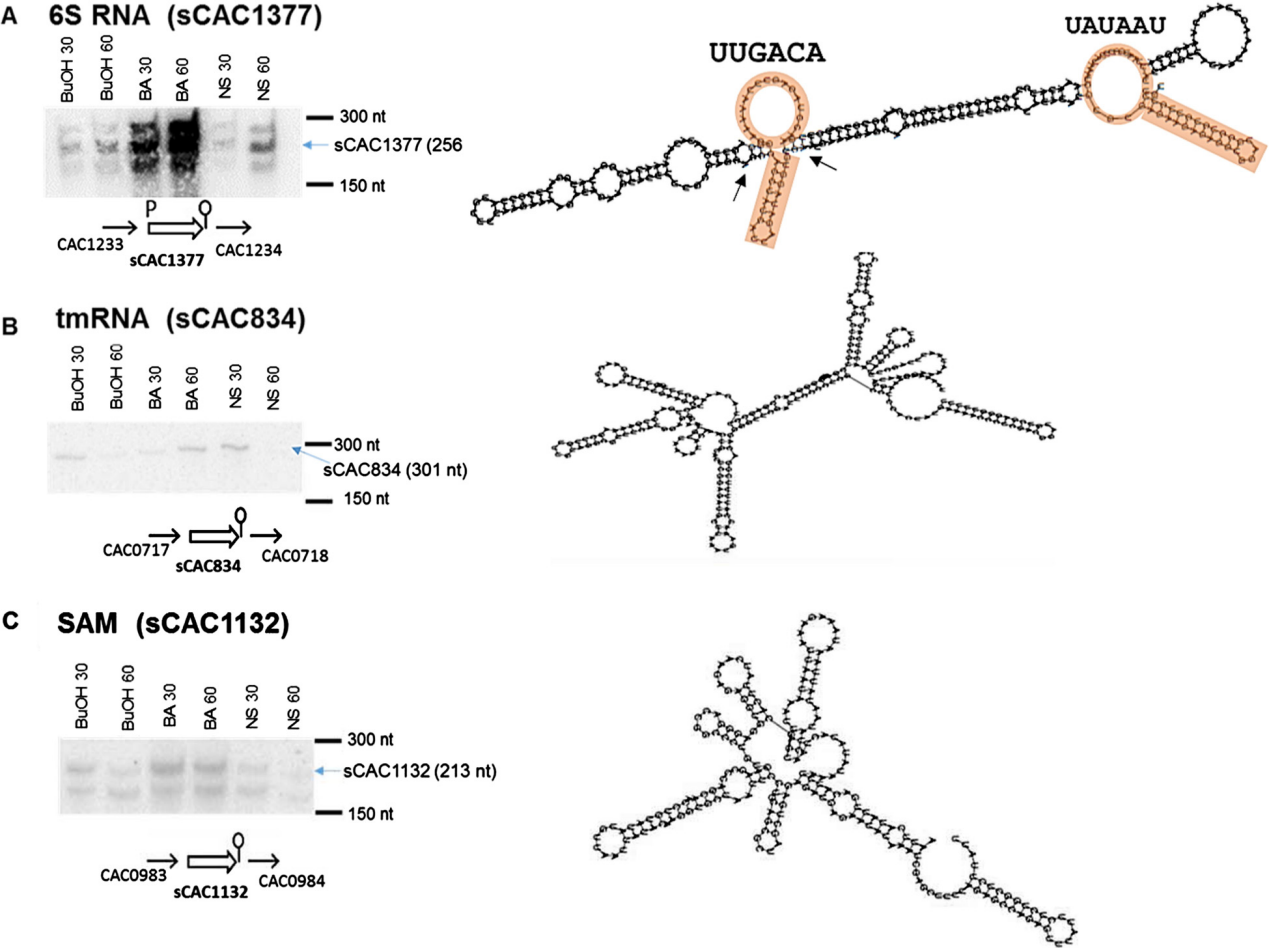
**Validation of sRNA expression by Northern analysis. (A)** 6S RNA; **(B)** tmRNA; &**(C)** SAM. The right arrow ( →) and left arrows (←) indicate the positive or negative orientation of the genes adjacent to the sRNA, while the orientation of the sRNA is represented by the double lined arrow. The symbol P, upstream of the sRNA represents the presence of a promoter region corresponding to one of the sigma factors (A, G, E & F) and the Ϙ symbol at the 3’ downstream end of the sRNA represents the rho independent terminator. The 6S RNA secondary structure shows the conserved asymmetric bubbles with G-C pairs at the end (arrows) (see text for details). BuOH- butanol, BA- butyrate, NS- no stress, 30 & 60 min post stress.

Among the differentially expressed sRNAs under metabolite stress described above, 31 have been previously validated by Northern and/or Q-RT-PCR analysis [40]. Here, we used Northern analysis (using single-stranded DNA probes to identify the strand specificity of the sRNA [34]) to examine the patterns of expression of a select number of differentially-expressed sRNAs. Selection was based on potential relevance to metabolite stress response (see below), but also on the ability to design probes, which requires that sRNAs have high GC content or GC rich regions. *tmRNA* (sCAC834), when analyzed by Northern blot, resulted in a single prominent band of ca. 300 nts. Northern blots of *6S* (sCAC1377) and *S-box* (SAM, sCAC1132) (Figure 4) revealed multiple bands indicating possible post-transcriptional processing by enzymes such as RNaseP, as has been reported [44].

**Figure 5:**
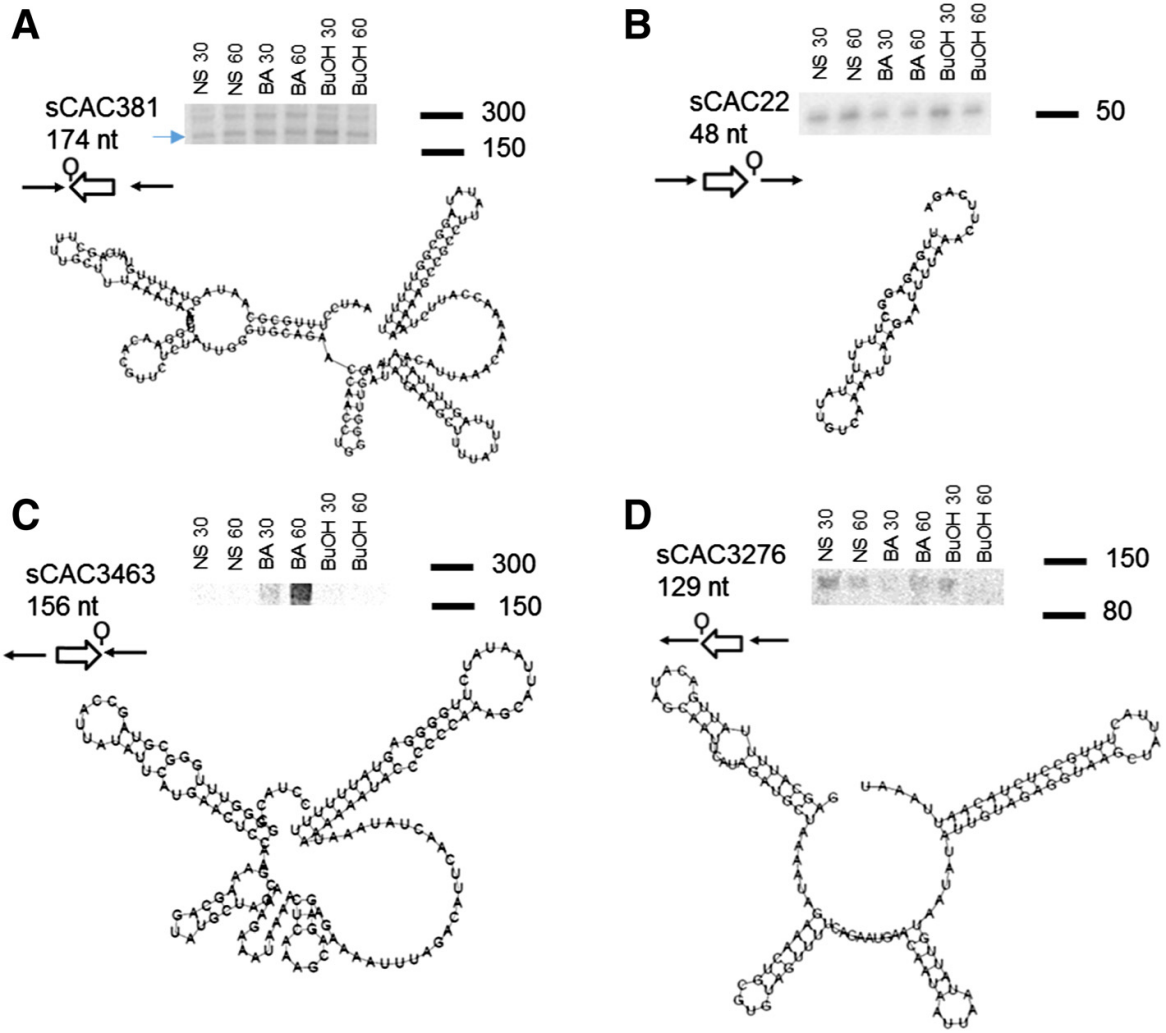
**Northern analysis of select, newly identified sRNAs and their predicted secondary structure. (A)** sCAC381; **(B)** sCAC22; **(C)** sCAC346; &**(D)** sCAC3276. BuOH- butanol, BA- butyrate, NS- no stress, 30 & 60 min post stress.

The sRNA predicted on INTEROP0218 (sCAC381 - 174 nt - predicted length), INTEROP0009 (sCAC22 - 48 nt - predicted length), INTEROP1858 (sCAC3276 - 129 nt - predicted length) and INTEROP1958 (sCAC3463 - 156 nt - predicted length) were successfully validated as being metabolite-stress responsive, with experimentally-estimated sizes (Figure 5) consistent with computational predicted lengths. Northern analysis of sCAC381 revealed two bands (~300 bp and ~174 bp), indicating possible RNA processing or two transcriptional start sites (TSS for the larger transcript may be located upstream of the regular TSS, but this needs to verified using either strand specific sequencing or 5’RACE).

### Patterns of expression: hierarchical clustering of sRNA expression under metabolite stress

**Figure 6:**
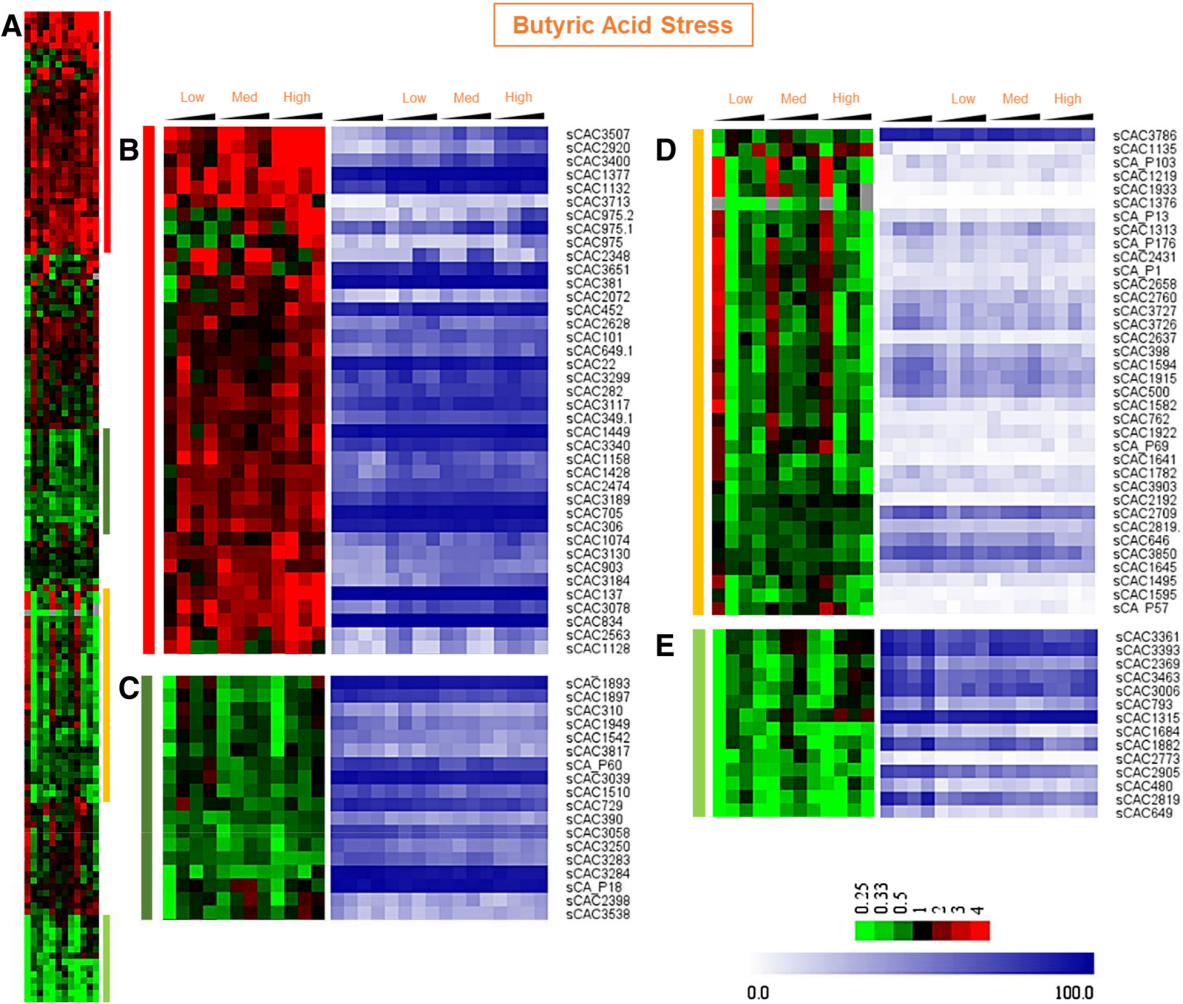
**Hierarchical clustering & expression profiles of sRNAs during butyric acid stress.** The expression profile is presented as the ratio of the normalized read counts under butyric acid stress against the corresponding time point in the no stress control. **(A)** Hierarchical clustering of the 159 sRNAs. Colored vertical bars represent expanded views of the regions on the right. **(B)** higher expression during butyric acid stress **(C)** weakly downregulated sRNAs **(D)** lower expression with delayed downregulation and **(E)** strongly downregulated genes. The blue plots show the expression/abundance ranking of each sRNA with respect to others as a percentile between 0 and 100.

Expression patterns under metabolite stress of the 159 sRNAs were compared against the non-stressed control cultures (pair-wise & point-by-point) and analyzed using hierarchical clustering. Both butyrate and butanol stress data displayed distinct clusters. Butyrate stress data resulted in four clusters. The 1st, “red”, cluster (Figure 6B) represents sRNAs that were expressed consistently higher compared to the control. The 2^nd^ “green” cluster (Figure 6C) consists of weakly downregulated sRNAs. The 3^rd^ cluster (Figure 6D) contained sRNAs that were downregulated with a small delay post-stress. The 4th cluster (Figure 6E) contains sRNAs showing a stronger (> 4.0 fold) downregulation at all three levels of butyrate stress. The blue plots display the level of relative expression (intensity ranking) among all sRNAs [45], and combined with the differential expression heat maps, provide a more accurate assessment of temporal patterns in differential expression and strength of expression. The sRNAs in the 1^st^, 2^nd^ and 4^th^ cluster show overall higher expression levels compared to the sRNAs of the 3^rd^ cluster.

**Figure 7:**
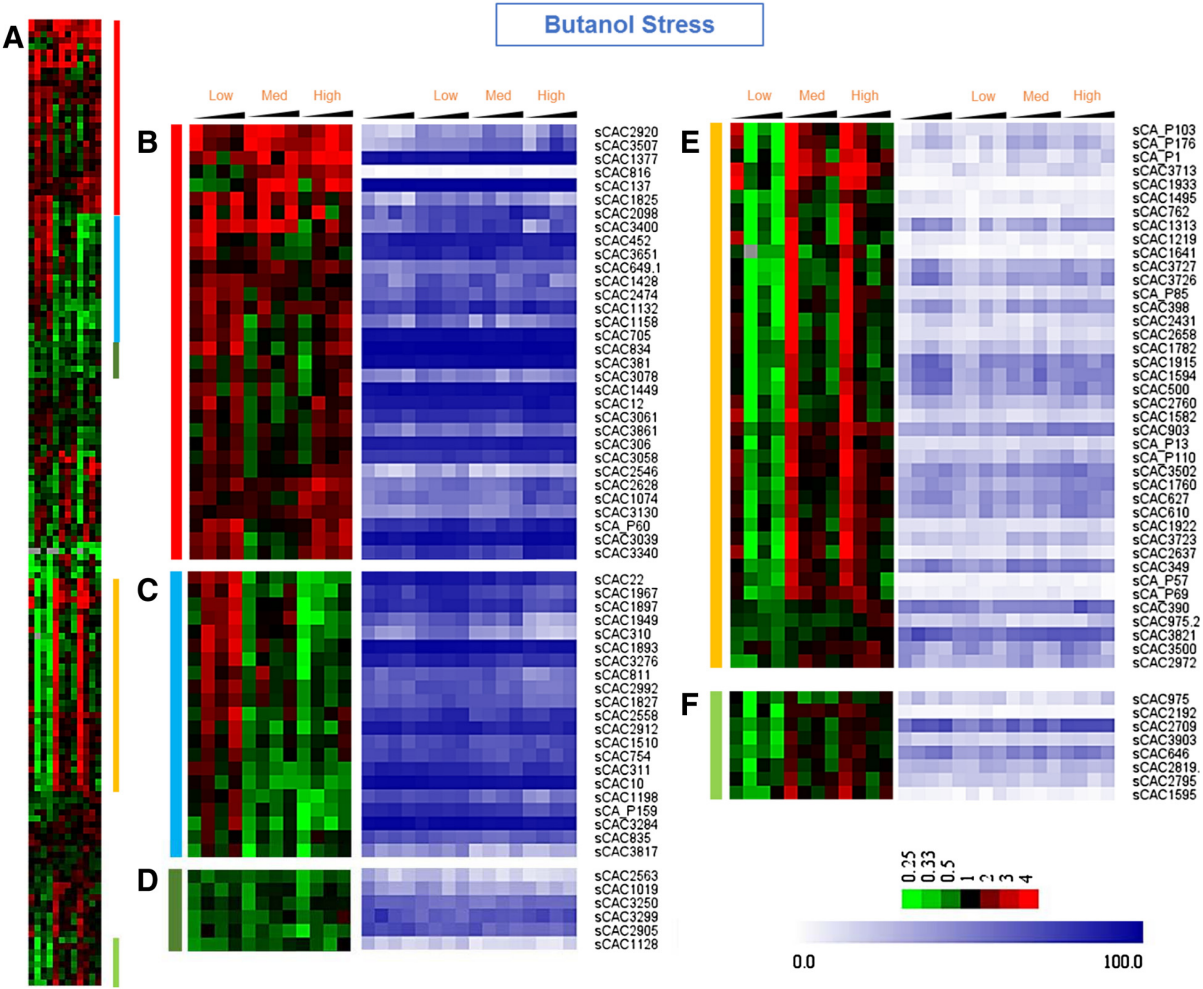
**Hierarchical clustering & expression profiles of sRNAs during butanol stress.** The expression profile is presented as the ratio of the normalized read counts under butanol stress against the corresponding time point in the no stress control. **(A)** Hierarchical clustering of the 159 sRNAs. Colored vertical bars represent expanded views of the regions on the right; **(B)** higher expression during butanol; **(C)** higher expression in low butanol stress **(D)** weakly downregulated sRNAs **(E)** and **(F)** downregulation only in low butanol stress. The blue plots show the expression/abundance ranking of each sRNA with respect to others as a percentile between 0 and 100.

sRNA expression under butanol stress also resulted in distinct, but more complex clusters. The 1^st^ cluster represented mostly upregulated sRNAs (Figure 7B). The 3^rd^ small cluster contained consistently downregulated sRNAs (Figure 7D). The remaining three clusters displayed a more complex pattern. The 2^nd^ cluster (Figure 7C) contained upregulated sRNAs only at low levels but not at medium or high levels of butanol stress. The 4^th^ and 5^th^ clusters (Figure 7E & 7 F) consisted of sRNAs that were downregulated at low levels of butanol stress, but not consistently so for medium or high levels of stress. The newly identified sRNAs, sCAC3400 (INTEROP1928), sCAC3507 (INTEROP1985) and sCAC2920 (INTEROP1658) were found to be upregulated under both stress conditions (Figures 6B & 7B), and these sRNAs had relatively stronger upregulation under butyrate stress than under butanol stress. Typically most target mRNAs of the *trans* sRNAs are located at a distant and different location on the genome. For example, the sRNAs ArcZ, DsrA, RprA and OxyS target the stress specific sigma factor RpoS in *E. coli* despite being located at different loci on the genome [24,25]. The differential expression of the sRNAs and their neighboring genes was analyzed by pairwise comparison of the no stress control sample against the three different levels of butanol or butyrate stress, (DEseq, p-value ≤ 0.05). Our analysis found very poor correlation between the differential expression of sRNAs and the neighboring genes (data not shown).

**Figure 8:**
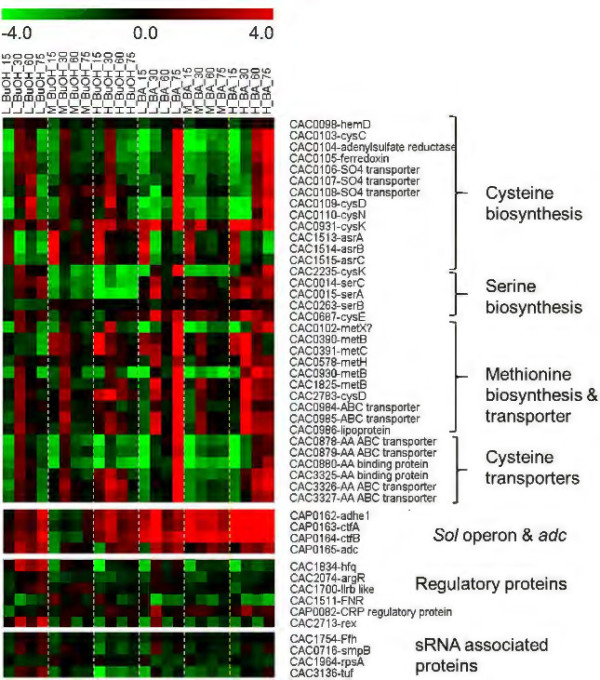
**Expression profile of genes involved in sulfur amino acid metabolism; part of the *sol *** **operon; and genes coding for Hfq and other regulatory proteins (see text for details).**

The clustered data were analyzed to identify shared regulatory elements, such as promoter sequences and transcription factor binding sites (TFBS) upstream of the sRNAs in the same cluster. Upstream regions of the sRNAs were scanned for putative promoter sites using *B. subtilis* position specific scoring matrices (PSSM) in the patser program within SIPHT [15] and the prokaryotic promoter prediction (PPP) tool for *Lactococcus* binding sites [46]. *B. subtilis* is the model Gram^+^ organism, while the (also Gram^+^) *Lactococcus* model was used as it has a more similar G + C content (35%) to *C. acetobutylicum* (29%). Using the *B. subtilis* model, we predicted that 52 of the 159 sRNAs (Additional file 1 & [40]) contain putative σ^A^, σ^E^, σ^F^ and σ^G^ promoters. Using the PPP webtool led to the identification of previously known stress-related motifs. Specifically, we analyzed two upregulated sRNA clusters: B1 (sCAC3507 to sCAC3713, Figure 6B) and B2 (sCAC3184 to sCAC1128, Figure 6B); and two clusters containing downregulated sRNAs: C (Figure 6C) and E (Figure 6E). Motifs for σ^A^, the house-keeping sigma factor, were identified in the upstream regions for most of the sRNAs analyzed. In addition to σ^A^, the upstream regions of the four clusters were enriched in binding motifs for σ^B^ (the general-stress response sigma factor in *B. subtilis*; however no σ^B^ ortholog has been identified in *C. acetobutylicum* or any other *Clostridium* organism [47]) and transcriptional factor binding sites (TFBS) for transcriptional factors such as FlpAB (the FNR family transcriptional regulator – which has two *C. acetobutylicum* ortholog genes, CAC1511 & CAP0082) [48,49], Llrb (two component system response regulator – with one *C. acetobutylicum* ortholog gene, CAC1700), Ahrc (arginine repressor – one *C. acetobutylicum* ortholog gene, CAC2074, coding for ArgR) (Foster, 2004) and Rex (redox sensing transcriptional repressor – one *C. acetobutylicum* ortholog gene, CAC2713) [50]. These proteins/transcriptional regulators (Figure 8) and their regulons have been identified to be part of oxidative stress response in some, at least, prokaryotes, and this might explain the presence of these motifs on sRNA promoters differentially expressed under butyrate stress, which is frequently similar to oxidative-stress response [35]. Identification of regulatory elements in the differentially expressed sRNA clusters B1, B2, C and E (Figure 6) reveal the presence of both general stress responsive elements (σ^B^) and the more specific oxidative stress response regulators (FNR, ArgR and Rex) supports the clustering of co-regulated stress responsive sRNAs.

### Hfq binding motifs on clostridial sRNAs

In *E. coli* and a few other prokaryotes, it has been shown that activity of several sRNAs (and notably of many trans-acting sRNAs) requires the assistance of, or is enhanced by, the hexameric RNA chaperone Hfq [51-55]. Thus, we wanted to examine which of the 159 sRNAs in *C. acetobutylicum* might be Hfq targets, and if these putative targets might be responsive to metabolite stress.

sRNAs co-immunoprecipitated with Hfq contain the signature Hfq-binding motif and are designated as Hfq-associated sRNAs [11,12,55,56]. This binding motif was discovered largely based on the *E. coli* sRNAs, but appears to be valid in other organisms [5,21,51,52,57] since the Hfq protein is well conserved among many prokaryotes. A structural CBLAST of the annotated *C. acetobutylicum* Hfq (CAC1834) with the two Hfq crystal structures, one from *E. coli*[58] (3GIB_B) and the other from *S. aureus*[54] (1KQ1_H), showed conservation in the sequence and the secondary structure of the Hfq monomeric unit (Additional file 3: Figure S1). Thus, we hypothesized that the binding motif of Hfq on sRNAs in *E. coli* might be preserved on sRNAs from *C. acetobutylicum*. This Hfq binding motif is characterized by U-rich regions, specifically a poly-U tail at the 3’ end of the sRNA (downstream of the Rho independent terminator), and the U-rich or the AU-rich region upstream of the rho-independent terminator or other secondary hairpin structure; the 5’ region of the sRNA is involved in (non-perfect) base-pairing with the target mRNA [52,53,55]. Using this model, we identified 65 potential Hfq-associated sRNAs in *C. acetobutylicum*. Among these 65 sRNAs, 20 sRNAs belonged to the 46 newly identified sRNA from the deep sequencing data (Additional file 4). We clustered these putative 65 Hfq-associated sRNAs and found most of them to be differentially expressed under both butanol and butyrate stress (Additional file 5: Figure S2). The Hfq gene (CAC1834) was found to be mildly differentially expressed (upregulated) only under butanol stress.

Identification of the putative Hfq binding module on 65 sRNAs may prove useful for deconvoluting the stress-responsive regulatory network in *Clostridia*, since the unstructured 5’ region of sRNAs that are targets of Hfq contains information that can be possibly used to identify potential target mRNAs of these sRNAs [59].

### Differentially expressed sRNAs that can be related to physiological events of the metabolite-stress response: *SRP RNA*, *6S* RNA, *tmRNA*, SAM RNA and *solB* (sCAP_176)

The data presented above showing a large number of sRNAs exhibiting differential expression under metabolite stress provides strong evidence that sRNAs are an integral part of the clostridial stress response system. While the detailed action of these sRNAs remains to be elucidated, there are several sRNAs whose action can be readily related to the phenotypic response of these cells to metabolite stress affecting various metabolic pathways as previously shown [35,37,39,42,60] and further confirmed by the present set of RNA-seq data as well as the accompanying large set of new microarray and proteomic data [61].

Both butanol and butyrate stress affect membrane physiology and homeostasis by reducing the transmembrane electrochemical potential and proton gradient (ΔpH) [33,34,62]. Bacteria respond to the toxicity of these metabolites by altering the membrane composition by increasing the percentage of saturation in the lipid tails and also by incorporating various integral membrane and transport proteins [63]. We have previously shown that the signal recognition particle (SRP) system and upregulation of several membrane proteins are apparently important in imparting butyric-acid tolerance [34]. The SRP, which consists of the SRP RNA and the Ffh protein, recognizes a motif on mRNAs coding for membrane proteins and, thus, transports the corresponding ribosomes to the membrane to synthesize the targeted proteins [64]. In this light, upregulation of the *4.5S SRP-RNA* (Figures 6 & 7) is consistent with its role in the biosynthesis and localization of membrane proteins and the role of membrane proteins in metabolite stress response and tolerance [34]. The Ffh gene is not differentially expressed under metabolite stress (Figure 8), thus further supporting its role as a housekeeping protein.

*C. acetobutylicum*, like other *Clostridium* organisms and most prokaryotes, reorganizes its transcriptional and translational machineries during the transition from exponential to stationary phase of growth and under stress conditions [35,37,38,42,45,47,60,65]. Downregulation of non-essential transcripts and overexpression of different transcript sets requires a quick turnover in the engagement of sigma factors. The 6S RNA (also known as SsrS RNA) has been shown to negatively regulate the transcripts under the control of the major sigma factor σ^70^ in *E. coli* and *B. subtilis* (where it is better known as σ^A^) during the stationary phase of growth by interacting with the RNA polymerase holoenzyme [66]. 6S RNA has been found to be important in cell survival under stress in both *E. coli* and *B. subtilis*[67,68]. We have previously shown that the 6S RNA in *C. acetobutylicum,* which displays the conserved secondary structure (Figure 4A) of an asymmetric bubble [69], is expressed at high levels [40]. Its Northern blot (Figure 4A) confirms its strong expression and displays multiple bands, which correspond to distinct processed RNA forms as in other prokaryotes [66,69]. In contrast to previously reported 6S RNAs displaying two sRNA forms of distinct size, here the 6S sRNA displays three bands (Figure 4A). 6S RNA acts as a template for binding of σ^70^, and is thus capable, when upregulated, of titrating σ^70^ thus leading to downregulation of genes under σ^70^ control. This stress responsive role of 6S RNA has been established in *E. coli*[70,71], and our data support that it has a similar role in *C. acetobutylicum*. It is notable that the 6S sRNA here contains the two characteristic central bubbles with a short stem loop attached [72]. The two components of the σ^A^ (the σ^70^ in *C. acetobutylicum*) binding motif (UUGACA [-35] & UAUAAU [-10], which corresponds to the DNA motif TTGACA and TATAAT) are found to be perfectly preserved, one on each of the central asymmetric bubbles (Figure 4A), thus apparently regulating the transcriptional responses to metabolite and other stresses. It is interesting to note that in *Legionella pneumophila*, 6S RNA was found to regulate the expression of secretion system effectors, and stress response proteins such as GroES and RecA [73]. As discussed, the GroESL system is one of the most upregulated HSP systems under a broad spectrum of stresses in *Clostridium* organisms.

The transfer-messenger RNA (tmRNA or SsrA RNA, which has both tRNA- and mRNA like properties) together with 3 proteins (small protein B [SmpB], elongation factor Tu [EF-Tu], and ribosomal protein S1) forms the tmRNP complex. The tmRNP complex is involved in the quality- control, so-called trans-translation process, recycling stalled ribosomes and facilitating the degradation of aberrant proteins and mRNAs [74,75]. Trans-translation is especially important in the transition between growth phases and under stress conditions [75-77], whereby many ribosomes may stall on damaged or partially degraded mRNAs. In this context, the stress-induced upregulation (Figures 4B, 6 & 7) of the *C. acetobutylicum* tmRNA (sCAC834) confirms its role in the quality-control process of trans-translation. It is worth noting that tmRNA is one of the most highly expressed sRNAs in these experiments (blue plots of Figures 6 & 7), further confirming its critical roles for the trans-translation process under stress. Of note, none of the three proteins (CAC0716 – smpB, SsrA-binding protein; CAC1964 – rpsA, 30S ribosomal protein S1; & CAC3136 – tuf, elongation factor Tu) of the tmRNP complex appear to be differentially expressed under stress (Figure 8), thus suggesting that the tmRNA upregulation is controlling the trans-translation process under stress. This is the first experimental evidence for the expression and role of a tmRNA in a *Clostridium* organism. Deletion of tmRNA in *Streptomyces coelicolor* was shown [78,79] to affect the translation of proteins that play a vital role in survival such as cell-cycle and stress proteins including the major HSP protein DnaK, a protein universally engaged in the stress response of *Clostridium* organisms as already discussed.

S-box (SAM) and T-box riboswitches regulate the expression of genes involved in the metabolism of cysteine and methionine in *C. acetobutylicum* and are typically found adjacent to the genes involved in sulfur amino acid metabolism [80]. S-box, which is dependent on the concentration of s-adenosyl methionine (SAM), has been shown to regulate the expression of genes in methionine metabolism through transcriptional anti-terminator systems [80]. In *C. acetobutylicum*, genes involved in sulfur metabolism were found to be upregulated (Figure 8) during high levels of acid stress. We note that an earlier study from our lab had reported that under acid stress, the genes involved in cysteine, methionine and serine metabolism were downregulated [35]; this difference between the two studies can be attributed to the role of proton concentration since in this present study, in contrast to the earlier one, we used pH control in the fermentation experiments.

Solventogenesis in *C. acetobutylicum* is controlled by the pSOL1-megaplasmid borne genes (*adhE1(aad)-ctfA-ctfB*) of the *sol* operon and the convergent monocistronic *adc* operon [81,82]. Expression of the *sol* operon is dependent on Spo0A [83] but other genes are also involved in regulating its expression through a long 5’ UTR, which appears like a good target for sRNA regulation. *solB* (sCAP_176), located just upstream of the *sol* operon, has been identified as the putative repressor of the *sol* operon and thus of solventogenesis [84]. Although expressed at very low levels (Figures 6 & 7), *solB* appears to be a very potent repressor: upon *solB* inactivation (originally achieved by inactivating the adjacent gene CAP0161 [85]), solvent formation starts earlier and leads to considerably higher levels of solvents [86]. Thus, *solB* downregulation promotes *sol* mRNA expression and solvent production, and vice versa. Here, we found that *solB* is downregulated (Figure 6D) under butyrate stress, except for the first time point (15 min post stress). Accordingly, the *sol*-operon genes display a strong upregulation pattern (Figure 8). Butanol stress leads to a more complex pattern of *solB* expression (Figure 7E), thus leading to a largely opposite expression of the *sol*-operon genes, except for the first two time points of the high butanol stress (Figure 8). Schiel et al. (2010) reported a putative antisense binding of the *solB* repressor to the upstream region of the *sol* operon [87] (Additional file 6: Figure S3).

## Conclusions

The goal of this study was to identify sRNAs that respond to butanol and/or butyrate stress, and also general stress, since, as we discussed, it was previously shown that toxic-chemical stress in *Clostridium* organisms engages both the general HSP systems as well as specialized systems. One can logically argue that the sRNAs that are differentially expressed under both butanol and butyrate stress would belong to the general stress response. In this sense, the putative roles of *SRP RNA*, *6S RNA*, *tmRNA* and *SAM RNA* are part of the general stress response, but perhaps *solB* belongs to the specialized stress response (Figures 6, 7 & Additional file 7: Figure S4). The metabolite-stress sRNome in *C. acetobutylicum* was investigated using deep RNA sequencing, in combination with computational analyses. 46 novel sRNAs were identified. The sRNA expression patterns under different levels of butanol and butyrate stress strongly support a role of many sRNAs in orchestrating stress-related cellular changes to deal with the complex, pleiotropic effects of the toxic metabolite stress. This is further supported by the fact that 7.5% of the RNA-seq reads map to non-annotated IOR and IGR of the genome. This is the first comprehensive study of genome-scale expression of sRNAs in a *Clostridium* or any organism under metabolite stress. Use of extensive temporal RNA-seq data in combination with computational predictions and Northern-based assays are essential in reaching robust outcomes in identifying previously unexplored sRNAs. These data can be used for understanding the role of sRNAs in regulating growth and metabolism thus aiming to provide a more comprehensive understanding of the regulatory network of the cell, and how that network can be engineered for practical applications to produce chemicals and fuels or for remediation processes.

## Methods

### Strain and growth conditions

Three biological replicate cultures of *C. acetobutylicum* ATCC 824 were carried out in pH- controlled (pH > 5) batch fermentations in 4 L bioreactors (Bioflow II and 110, New Brunswick Scientific, Edison, NJ, USA) in a defined clostridial growth media [61]. The cultures were stressed with butanol (30 mM, 60 mM and 90 mM) and butyric acid (30 mM, 40 mM and 50 mM) at mid-exponential phase of growth at an OD of 1.0 and were sampled at 4 different time points: 15 min, 30 min, 60 min and 75 min post stress. Parallel cultures (n = 3) that were exposed to neither stress were used as the non-stress controls.

### RNA isolation and construction of cDNA libraries for RNA-seq

Samples for RNA isolation were collected by centrifugation at 5000 rpm at 4°C for 10 min and the pellets were stored at -80°C. RNA isolation was carried out using the Qiagen’s miRNeasy Mini kit [45]. After RNA extraction, mRNA and sRNA were enriched by using Microbe Express kit from Ambion® kit as per the manufacturer’s protocol. The Ovation Prokaryotic RNA-Seq System (NuGEN® Technologies, Inc, San Carlos, CA) was used to synthesize cDNA from 500 ng of enriched RNA. In brief, 2 μL of first primer mix was added to the 500 ng of the RNA and incubated at 65°C for 5 min. Later, 10 μL of the master mix (first strand buffer and enzyme) were added to the above reaction for first strand synthesis followed by the purification of the first strand cDNA using the QiaQuick PCR purification kit (QIAGEN, Inc. Valencia, CA). The last step of cDNA synthesis was synthesizing the 2^nd^ strand, which was then purified using the Minelute Reaction clean up kit (QIAGEN®) and eluted in 10 μL of elution buffer. The elution buffer was used to make up the volume of the cDNA to 50 μL. The resulting 50 μL of cDNA was used to construct libraries using the TruSeq DNA Sample preparation kit (Illumina®, San Diego, CA). In brief, the cDNA underwent end repair, 3’ end adenylation, adapter ligation and enrichment. Clean up of DNA fragments after each process were carried out using AMPure XP Beads. The fragment length of the libraries was checked using a Bioanalyzer before loading onto HiSeq 2000.

### RNA sequencing and data analyses

Deep sequencing was performed using Illumina's HiSeq 2000 with a read length of 50 bp, generating individual library sequence files. Sequence files were processed to remove barcodes, trim adapters, and count read abundances using a set of custom perl, python, and MySQL scripts. Reads were mapped to the *C. acetobutylicum* genome using Tophat [88]. Gene annotations were downloaded from NCBI, and predicted sRNA regions were included from Chen *et al.*[40]. Differential expression analysis of sRNAs was performed using DESeq, part of the R Bioconductor package [89]. Differentially expressed sRNAs were determined at a p-value ≤ 0.05, for a pairwise comparison between the control library set and any of the six stress groups (low, medium, and high butanol and low, medium, and high butyrate). Interoperonic regions were defined using previously predicted operons [43]. The data was submitted to Gene Expression Omnibus (GEO) and can be accessed with the accession number GSE48349.

### Prediction of novel sRNAs using RNA-seq data

The IORs used in this study were the same as those identified and used by Chen et al. for predicting the 113 sRNAs in *C. acetobutylicum*[40]. Identification of new sRNAs was based on selecting interoperonic regions (IORs) with a minimal read count of 50. This metric was assigned based on the analysis of the RNA-seq data for mRNAs and sRNAs (Figure 1). Only IORs that met the criteria of a minimal read count were considered for further analysis. Computational prediction of sRNAs was performed using SIPHT based on the comparative analysis of the 21 *Clostridium* genomes in NCBI. The previously identified 113 sRNAs [40] were removed for predicting novel sRNAs. Thus, IORs expressed at a minimal read count of 50 and were also computationally predicted to contain sRNAs in the expressed IORs, were manually curated to eliminate false positives. False positives were defined as IORs, which had predominant expression only from the untranslated region (UTRs) of the neighboring genes, even though sRNAs were computationally predicted in those regions. Identification of false positives was carried out using a custom web viewer (generated using custom PHP scripts) by visually analyzing RNA-seq data.

### Northern analysis

Northern analysis of select sRNAs was performed as described previously using single stranded oligo DNA probes [40]. The probes used in Northern analysis are listed in Additional file 2: Table S2. For each lane, 10 μg of total RNA was loaded in a 5% precast polyacrylamide Ready Gel TBE-urea (Bio-Rad, Hercules, CA), and was electrophoretically resolved along with molecular markers of single stranded RNA ranging from 50 nt to 1000 nt (New England Biolabs, Ipswich, MA). Following electrophoresis, the RNA was transferred to a BrightStar®-Plus positively charged nylon membrane (Ambion). Probes were labeled with ATP [γ^32^P] using Optikinase (USB, Cleveland, OH) and the unincorporated radioactive material was removed using Micro Bio-Spin Column (Bio-Rad, Hercules, CA). The prehybridization and hybridization of the membrane with labeled oligo probes was carried using the ULTRAhyb hybridization solution (Ambion) at 42°C.

### Promoters and Rho-independent terminators of sRNAs

Promoter prediction in the upstream region of the sRNAs were carried out using PSSMs of *B. subtilis* promoter consensus sequences [15]. For the *Lactococcus* promoters, we used the promoter HMM model from the PPP tool [46]. Rho independent terminators were predicted using RNAmotif [90], Erpin [91] and Findterm (http://www.softberry.com). Secondary structures of sRNAs were predicted using Vienna RNAfold [92,93].

### sRNA nomenclature

The sRNA nomenclature for the newly identified sRNAs was done in the same manner as described previously [40].

## Abbreviations

SIPHT: sRNA identification protocol using high-throughput technologies; ABE: Acetone-bytanol-ethanol; Q-RT-PCR: Quantitative reverse transcriptase polymerase chain reaction; IOR: Interoperonic region; IGR: Intergenic region; ORF: Open reading frame; UTR: Untranslated region; PSSM: Position specific scoring matrix; HMM: Hidden Markov model; FNR: Ferredoxin-NADP^+^ reductase; AhrC/ArgR: Arginine repressor; Hfq: Host factor-I protein from Q8 bacteriophage; TFBS: Transcriptional factor binding site; SRP: Signal recognition particle; tmRNA: Transfer and messenger RNA; SAM: S-adenosyl methionine; adhe: Alcohol/aldehyde dehydrogenase 1; ctf: Co-enzyme A transferase; adc: Acetoacetate decarboxylase; SmpB: Small protein B; EF-Tu/Tuf: Elongation factor Tu; rpsA: Ribosomal protein A.

## Competing interests

The authors declare that they have no competing interests.

## Authors’ contributions

KPV and SWJ carried out the fermentations and RNA isolations. KPV carried out Northern analysis and drafted the manuscript. SGK constructed the libraries for RNA-seq. KPM processed the data and built the web browser. KPV and KPM analysed the data. KPV and MTR carried out the identification of new sRNAs and other computational analyses. BCM and ETP helped with data analysis and interpretation, and participated in the manuscript preparation. ETP conceived the study, participated in its design and coordination. All authors read and approved the final manuscript.

## Supplementary Material

Additional file 1 Detailing the information on the sequenced libraries and the list of newly identified 46 sRNAs. Click here for file

Additional file 2: Table S1 Differential expression of annotated mRNAs of *C. acetobutylicum*. Pair-wise and point by point by comparison of each stress level to the no stress control using DEseq at a p-value ≤ 0.05. **Table S2**: Probes sequences used for Northern analysis. Click here for file

Additional file 3: Figure S1 CBLAST of the *C. acetobutylicum* hfq (CAC1834, gi_15895109) with the Hfq from *E. coli* (3GIB_B), reveals conservation in the secondary structure on the Hfq monomeric unit. (A) The α-β_1-5_ structural unit can be found to be conserved. (B) The corresponding conservation in the protein sequence is displayed below. Click here for file

Additional file 4 Containing the Hfq binding model. Click here for file

Additional file 5: Figure S2 Hierarchical clustering of the 65 sRNAs belonging to the Hfq constellation (see text for details). Click here for file

Additional file 6: Figure S3 Putative antisense binding of *solB* to the *sol* (*adhE1-ctfA-ctfB*) operon. Click here for file

Additional file 7: Figure S4 Hierarchical clustering of 159 sRNAs under both butanol and butyric acid stress. Click here for file

## References

[B1] Argaman L Hershberg R Vogel J Bejerano G Wagner EGH Margalit H Altuvia S Novel small RNA-encoding genes in the intergenic regions of Escherichia coli Curr Biol 2001 14 12 941 950 10.1016/S0960-9822(01)00270-6 11448770

[B2] Brownlee GG Sequence of 6s RNA of *E. coli* Nature-New Biol 1971 14 5 147 10.1038/newbio229147a0 4929322

[B3] Hindley J Fractionation of 32P-labelled ribonucleic acids on polyacrylamide gels and their characterization by fingerprinting J Mol Biol 1967 14 1 125 136 10.1016/0022-2836(67)90248-3 4865141

[B4] Storz G Haas D A guide to small RNAs in microorganisms Curr Opin Microbiol 2007 14 2 93 95 10.1016/j.mib.2007.03.017

[B5] Lenz DH Mok KC Lilley BN Kulkarni RV Wingreen NS Bassler BL The small RNA chaperone Hfq and multiple small RNAs control quorum sensing in Vibrio harveyi and Vibrio cholerae Cell 2004 14 1 69 82 10.1016/j.cell.2004.06.009 15242645

[B6] Livny J Brencic A Lory S Waldor MK Identification of 17 Pseudomonas aeruginosa sRNAs and prediction of sRNA-encoding genes in 10 diverse pathogens using the bioinformatic tool sRNAPredict2 Nucleic Acids Res 2006 14 12 3484 3493 10.1093/nar/gkl453 16870723 PMC1524904

[B7] Shinhara A Matsui M Hiraoka K Nomura W Hirano R Nakahigashi K Tomita M Mori H Kanai A Deep sequencing reveals as-yet-undiscovered small RNAs in *Escherichia coli* BMC Genomics 2011 14 428 10.1186/1471-2164-12-428 21864382 PMC3175480

[B8] Irnov I Sharma CM Vogel J Winkler WC Identification of regulatory RNAs in Bacillus subtilis Nucleic acids research 2010 14 19 6637 6651 10.1093/nar/gkq454 20525796 PMC2965217

[B9] Mraheil MA Billion A Mohamed W Mukherjee K Kuenne C Pischimarov J Krawitz C Retey J Hartsch T Chakraborty T The intracellular sRNA transcriptome of Listeria monocytogenes during growth in macrophages Nucleic Acids Res 2011 14 10 4235 4248 10.1093/nar/gkr033 21278422 PMC3105390

[B10] Patenge N Billion A Raasch P Normann J Wisniewska-Kucper A Retey J Boisguerin V Hartsch T Hain T Kreikemeyer B Identification of novel growth phase- and media-dependent small non-coding RNAs in *Streptococcus pyogenes* M49 using intergenic tiling arrays BMC Genomics 2012 14 550 10.1186/1471-2164-13-550 23062031 PMC3542284

[B11] Sittka A Lucchini S Papenfort K Sharma CM Rolle K Binnewies TT Hinton JCD Vogel J Deep Sequencing Analysis of Small Noncoding RNA and mRNA Targets of the Global Post-Transcriptional Regulator, Hfq PLoS Genet 2008 14 8 e1000163. doi:10.1371/journal.pgen.1000163 10.1371/journal.pgen.1000163 PMC2515195 18725932

[B12] Sittka A Sharma CM Rolle K Vogel J Deep sequencing of Salmonella RNA associated with heterologous Hfq proteins in vivo reveals small RNAs as a major target class and identifies RNA processing phenotypes RNA Biol 2009 14 3 266 275 10.4161/rna.6.3.8332 19333007

[B13] Wilms I Overloper A Nowrousian M Sharma CM Narberhaus F Deep sequencing uncovers numerous small RNAs on all four replicons of the plant pathogen Agrobacterium tumefaciens RNA Biol 2012 14 4 446 457 10.4161/rna.17212 22336765 PMC3384568

[B14] Livny J Waldor MK Identification of small RNAs in diverse bacterial species Curr Opin Microbiol 2007 14 2 96 101 10.1016/j.mib.2007.03.005 17383222

[B15] Livny J Fogel MA Davis BM Waldor MK sRNAPredict: an integrative computational approach to identify sRNAs in bacterial genomes Nucleic Acids Res 2005 14 13 4096 4105 10.1093/nar/gki715 16049021 PMC1180744

[B16] Rivas E Eddy SR Noncoding RNA gene detection using comparative sequence analysis BMC Bioinformatics 2001 14 1 8 10.1186/1471-2105-2-8 11801179 PMC64605

[B17] Pichon C Felden B Intergenic sequence inspector: searching and identifying bacterial RNAs Bioinformatics 2003 14 13 1707 1709 10.1093/bioinformatics/btg235 15593399

[B18] Sridhar J Narmada SR Sabarinathan R Ou HY Deng ZX Sekar K Rafi ZA Rajakumar K sRNAscanner: A Computational Tool for Intergenic Small RNA Detection in Bacterial Genomes PloS One 2010 14 8 e11970. doi: 10.1371/journal.pone.0011970 10.1371/journal.pone.0011970 PMC2916834 20700540

[B19] Domenech P Honore N Heym B Cole ST Role of OxyS of Mycobacterium tuberculosis in oxidative stress: overexpression confers increased sensitivity to organic hydroperoxides Microbes Infect 2001 14 9 713 721 10.1016/S1286-4579(01)01422-8 11489419

[B20] Mey AR Craig SA Payne SM Characterization of Vibrio cholerae RyhB: the RyhB regulon and role of ryhB in biofilm formation Infect Immun 2005 14 9 5706 5719 10.1128/IAI.73.9.5706-5719.2005 16113288 PMC1231101

[B21] Davis BM Quinones M Pratt J Ding YP Waldor MK Characterization of the small untranslated RNA RyhB and its regulon in Vibrio cholerae J Bacteriol 2005 14 12 4005 4014 10.1128/JB.187.12.4005-4014.2005 15937163 PMC1151736

[B22] Gottesman S McCullen C Guillier M Vanderpool C Majdalani N Benhammou J Thompson K FitzGerald P Sowa N FitzGerald D Small RNA regulators and the bacterial response to stress Cold Spring Harb Symp Quant Biol 2006 14 1 11 10.1101/sqb.2006.71.016 10.1101/sqb.2006.71.016 17381274 PMC3592358

[B23] Negrete A Ng WI Shiloach J Glucose uptake regulation in *E. coli* by the small RNA SgrS: comparative analysis of *E. coli* K-12 (JM109 and MG1655) and *E. coli* B (BL21) Microb Cell Fact 2010 14 75 10.1186/1475-2859-9-75 10.1186/1475-2859-9-75 20920177 PMC2955591

[B24] Battesti A Majdalani N Gottesman S The RpoS-Mediated General Stress Response in Escherichia coli Annu Rev Microbiol 2011 14 189 213 10.1146/annurev-micro-090110-102946 21639793 PMC7356644

[B25] Mandin P Gottesman S Integrating anaerobic/aerobic sensing and the general stress response through the ArcZ small RNA EMBO J 2010 14 18 3094 3107 10.1038/emboj.2010.179 20683441 PMC2944060

[B26] Repoila F Majdalani N Gottesman S Small non-coding RNAs, co-ordinators of adaptation processes in Escherichia coli: the RpoS paradigm Mol Microbiol 2003 14 4 855 861 10.1046/j.1365-2958.2003.03454.x 12753181

[B27] Tracy BP Jones SW Fast AG Indurthi DC Papoutsakis ET Clostridia: the importance of their exceptional substrate and metabolite diversity for biofuel and biorefinery applications Curr Opin Biotechnol 2012 14 3 364 381 10.1016/j.copbio.2011.10.008 22079352

[B28] Fast AG Papoutsakis ET Stoichiometric and energetic analyses of non-photosynthetic CO2-fixation pathways to support synthetic biology strategies for production of fuels and chemicals Curr Opin Chem Eng 2012 14 4 380 395 10.1016/j.coche.2012.07.005

[B29] Morgan XC Tickle TL Sokol H Gevers D Devaney KL Ward DV Reyes JA Shah SA LeLeiko N Snapper SB Dysfunction of the intestinal microbiome in inflammatory bowel disease and treatment Genome Biol 2012 14 9 R79 10.1186/gb-2012-13-9-r79 23013615 PMC3506950

[B30] Alvarez-Ordonez A Mouwen DJM Lopez M Prieto M Fourier transform infrared spectroscopy as a tool to characterize molecular composition and stress response in foodborne pathogenic bacteria J Microbiol Meth 2011 14 3 369 378 10.1016/j.mimet.2011.01.009 21256893

[B31] Chowdhury R Sahu GK Das J Stress response in pathogenic bacteria J Biosci 1996 14 2 149 160 10.1007/BF02703105

[B32] Hassett DJ Cohen MS Bacterial Adaptation to Oxidative Stress - Implications for Pathogenesis and Interaction with Phagocytic-Cells FASEB J 1989 14 14 2574 2582 2556311 10.1096/fasebj.3.14.2556311

[B33] Nicolaou SA Gaida SM Papoutsakis ET A comparative view of metabolite and substrate stress and tolerance in microbial bioprocessing: From biofuels and chemicals, to biocatalysis and bioremediation Metab Eng 2010 14 4 307 331 10.1016/j.ymben.2010.03.004 20346409

[B34] Borden JR Jones SW Indurthi D Chen Y Papoutsakis ET A genomic-library based discovery of a novel, possibly synthetic, acid-tolerance mechanism in Clostridium acetobutylicum involving non-coding RNAs and ribosomal RNA processing Metab Eng 2010 14 3 268 281 10.1016/j.ymben.2009.12.004 20060060 PMC2857598

[B35] Alsaker KV Paredes C Papoutsakis ET Metabolite stress and tolerance in the production of biofuels and chemicals: gene-expression-based systems analysis of butanol, butyrate, and acetate stresses in the anaerobe Clostridium acetobutylicum Biotechnol Bioeng 2010 14 6 1131 1147 19998280 10.1002/bit.22628

[B36] Gaida SM Al-Hinai MA Indurthi D Nicolaou SA Papoutsakis ET Synthetic tolerance: three noncoding small RNAs, DsrA, ArcZ and RprA, acting supra-additively against acid stress Nucleic Acids Res 2013 14 10.1093/nar/gkt1651 10.1093/nar/gkt651 PMC3794604 23892399

[B37] Janssen H Grimmler C Ehrenreich A Bahl H Fischer RJ A transcriptional study of acidogenic chemostat cells of Clostridium acetobutylicum-Solvent stress caused by a transient n-butanol pulse J Biotechnol 2012 14 3 354 365 10.1016/j.jbiotec.2012.03.027 22537853

[B38] Alsaker KV Spitzer TR Papoutsakis ET Transcriptional analysis of spo0A overexpression in Clostridium acetobutylicum and its effect on the cell's response to butanol stress J Bacteriol 2004 14 7 1959 1971 10.1128/JB.186.7.1959-1971.2004 15028679 PMC374416

[B39] Tomas CA Beamish J Papoutsakis ET Transcriptional analysis of butanol stress and tolerance in Clostridium acetobutylicum J Bacteriol 2004 14 7 2006 2018 10.1128/JB.186.7.2006-2018.2004 15028684 PMC374415

[B40] Chen Y Indurthi DC Jones SW Papoutsakis ET Small RNAs in the genus *Clostridium* Ambio 2011 14 1 e00340 00310 10.1128/mBio.00340-10 10.1128/mBio.00340-10 PMC3025663 21264064

[B41] Nicolaou SA Gaida SM Papoutsakis ET Coexisting/Coexpressing Genomic Libraries (CoGeL) identify interactions among distantly located genetic loci for developing complex microbial phenotypes Nucleic Acids Res 2011 14 22 e152 10.1093/nar/gkr817 21976725 PMC3239195

[B42] Alsaker KV Papoutsakis ET Transcriptional program of early sporulation and stationary-phase events in Clostridium acetobutylicum J Bacteriol 2005 14 20 7103 7118 10.1128/JB.187.20.7103-7118.2005 16199581 PMC1251621

[B43] Paredes CJ Rigoutsos I Papoutsakis ET Transcriptional organization of the Clostridium acetobutylicum genome Nucleic Acids Res 2004 14 6 1973 1981 10.1093/nar/gkh509 15060177 PMC390361

[B44] Komine Y Kitabatake M Yokogawa T Nishikawa K Inokuchi H A Transfer-Rna-Like Structure Is Present in 10sa Rna, a Small Stable Rna from Escherichia coli Proc Natl Acad Sci USA 1994 14 20 9223 9227 10.1073/pnas.91.20.9223 7524073 PMC44784

[B45] Jones SW Paredes CJ Tracy B Cheng N Sillers R Senger RS Papoutsakis ET The transcriptional program underlying the physiology of clostridial sporulation Genome Biol 2008 14 7 R114 10.1186/gb-2008-9-7-r114 18631379 PMC2530871

[B46] Zomer AL Buist G Larsen R Kok J Kuipers OP Time-resolved determination of the CcpA regulon of Lactococcus lactis subsp cremoris MG1363 J Bacteriol 2007 14 4 1366 1381 10.1128/JB.01013-06 17028270 PMC1797362

[B47] Paredes CJ Alsaker KV Papoutsakis ET A comparative genomic view of clostridial sporulation and physiology Nat Rev Microbiol 2005 14 12 969 978 10.1038/nrmicro1288 16261177

[B48] Akhter Y Tundup S Hasnain SE Novel biochemical properties of a CRP/FNR family transcription factor from Mycobacterium tuberculosis Int J Med Microbiol 2007 14 6 451 457 10.1016/j.ijmm.2007.04.009 17702648

[B49] Kazakov AE Rodionov DA Price MN Arkin AP Dubchak I Novichkov PS Transcription Factor Family-Based Reconstruction of Singleton Regulons and Study of the Crp/Fnr, ArsR, and GntR Families in Desulfovibrionales Genomes J Bacteriol 2013 14 1 29 38 10.1128/JB.01977-12 23086211 PMC3536172

[B50] Wietzke M Bahl H The redox-sensing protein Rex, a transcriptional regulator of solventogenesis in Clostridium acetobutylicum Appl Microbiol Biotechnol 2012 14 3 749 761 10.1007/s00253-012-4112-2 22576944

[B51] Vogel J Luisi BF Hfq and its constellation of RNA Nat Rev Microbiol 2011 14 8 578 589 10.1038/nrmicro2615 21760622 PMC4615618

[B52] Otaka H Ishikawa H Morita T Aiba H PolyU tail of rho-independent terminator of bacterial small RNAs is essential for Hfq action Proc Natl Acad Sci USA 2011 14 32 13059 13064 10.1073/pnas.1107050108 21788484 PMC3156202

[B53] Papenfort K Bouvier M Mika F Sharma CM Vogel J Evidence for an autonomous 5 ' target recognition domain in an Hfq-associated small RNA Proc Natl Acad Sci U S A 2010 14 47 20435 20440 10.1073/pnas.1009784107 21059903 PMC2996696

[B54] Schumacher MA Pearson RF Moller T Valentin-Hansen P Brennan RG Structures of the pleiotropic translational regulator Hfq and an Hfq-RNA complex: a bacterial Sm-like protein EMBO J 2002 14 13 3546 3556 10.1093/emboj/cdf322 12093755 PMC126077

[B55] Ishikawa H Otaka H Maki K Morita T Aiba H The functional Hfq-binding module of bacterial sRNAs consists of a double or single hairpin preceded by a U-rich sequence and followed by a 3 ' poly(U) tail RNA 2012 14 5 1062 1074 10.1261/rna.031575.111 22454537 PMC3334693

[B56] Ikeda Y Yagi M Morita T Aiba H Hfq binding at RhlB-recognition region of RNase E is crucial for the rapid degradation of target mRNAs mediated by sRNAs in Escherichia coli Mol Microbiol 2011 14 2 419 432 10.1111/j.1365-2958.2010.07454.x 21219461

[B57] Brennan RG Link TM Hfq structure, function and ligand binding Curr Opin Microbiol 2007 14 2 125 133 10.1016/j.mib.2007.03.015 17395525

[B58] Link TM Valentin-Hansen P Brennan RG Structure of Escherichia coli Hfq bound to polyriboadenylate RNA Proc Natl Acad Sci USA 2009 14 46 19286 19291 10.1073/pnas.0905469106 19889981 PMC2773200

[B59] Beisel CL Updegrove TB Janson B Storz G Multiple factors dictate target selection by Hfq-binding small RNAs EMBO J 2012 14 8 1961 1974 10.1038/emboj.2012.52 22388518 PMC3343335

[B60] Schwarz KM Kuit W Grimmler C Ehrenreich A Kengen SWM A transcriptional study of acidogenic chemostat cells of Clostridium acetobutylicum - Cellular behavior in adaptation to n-butanol J Biotechnol 2012 14 3 366 377 10.1016/j.jbiotec.2012.03.018 22484128

[B61] Hou S Jones SW Choe LH Papoutsakis ET Lee KH Workflow for quantitative proteomic analysis of *Clostridia acetobutylicum* ATCC 824 using iTRAQ tags Methods 2013 14 3 269 276 10.1016/j.ymeth.2013.03.013 10.1016/j.ymeth.2013.03.013 23523702

[B62] Wang FQ Kashket S Kashket ER Maintenance of Delta pH by a butanol-tolerant mutant of Clostridium beijerinckii Microbiol-Sgm 2005 14 607 613 10.1099/mic.0.27587-0 15699209

[B63] Heipieper HJ Weber FJ Sikkema J Keweloh H Debont JAM Mechanisms of Resistance of Whole Cells to Toxic Organic-Solvents Trends Biotechnol 1994 14 10 409 415 10.1016/0167-7799(94)90029-9

[B64] Poritz MA Bernstein HD Strub K Zopf D Wilhelm H Walter P An *Escherichia coli* Ribonucleoprotein Containing 4.5s Rna Resembles Mammalian Signal Recognition Particle Science 1990 14 4984 1111 1117 10.1126/science.1701272 1701272

[B65] Honicke D Janssen H Grimmler C Ehrenreich A Lutke-Eversloh T Global transcriptional changes of Clostridium acetobutylicum cultures with increased butanol:acetone ratios N Biotechnol 2012 14 4 485 493 10.1016/j.nbt.2012.01.001 22285530

[B66] Cavanagh AT Sperger JM Wassarman KM Regulation of 6S RNA by pRNA synthesis is required for efficient recovery from stationary phase in E. coli and B. subtilis Nucleic Acids Res 2012 14 5 2234 2246 10.1093/nar/gkr1003 22102588 PMC3299989

[B67] Geissen R Steuten B Polen T Wagner R E. coli 6S RNA A universal transcriptional regulator within the centre of growth adaptation RNA Biol 2010 14 5 564 568 10.4161/rna.7.5.12969 20930516

[B68] Sharma UK Chatterji D Transcriptional switching in Escherichia coli during stress and starvation by modulation of Sigma 70 activity FEMS Microbiol Rev 2010 14 5 646 657 20491934 10.1111/j.1574-6976.2010.00223.x

[B69] Soutourina OA Monot M Boudry P Saujet L Pichon C Sismeiro O Semenova E Severinov K Le Bouguenec C Coppée J-Y Genome-Wide Identification of Regulatory RNAs in the Human Pathogen Clostridium difficile PLoS Genet 2013 14 5 e1003493 10.1371/journal.pgen.1003493 23675309 PMC3649979

[B70] Chae H Han K Kim KS Park H Lee J Lee Y Rho-dependent Termination of ssrS (6S RNA) Transcription in Escherichia coli: implication for 3' processing of 6S RNA and expression of downstream ygfA (putative 5-formyl-tetrahydrofolate cyclo-ligase) J Biol Chem 2011 14 1 114 122 10.1074/jbc.M110.150201 21036909 PMC3012964

[B71] Kim KS Lee Y Regulation of 6S RNA biogenesis by switching utilization of both sigma factors and endoribonucleases Nucleic Acids Res 2004 14 20 6057 6068 10.1093/nar/gkh939 15550566 PMC534622

[B72] Griffiths-Jones S Moxon S Marshall M Khanna A Eddy SR Bateman A Rfam: annotating non-coding RNAs in complete genomes Nucleic Acids Res 2005 14 D121 D124 15608160 10.1093/nar/gki081 PMC540035

[B73] Faucher SP Friedlander G Livny J Margalit H Shuman HA Legionella pneumophila 6S RNA optimizes intracellular multiplication Proc Natl Acad Sci USA 2010 14 16 7533 7538 10.1073/pnas.0911764107 20368425 PMC2867745

[B74] Keiler KC Biology of trans-Translation Annu Rev Microbiol 2008 14 133 151 10.1146/annurev.micro.62.081307.162948 18557701

[B75] Tadaki T Fukushima M Ushida C Himeno H Muto A Interaction of 10Sa RNA with ribosomes in Escherichia coli FEBS Lett 1996 14 3 223 226 10.1016/S0014-5793(96)01330-0 8985150

[B76] Felden B Himeno H Muto A McCutcheon JP Atkins JF Gesteland RF Probing the structure of the Escherichia coli 10Sa RNA (tmRNA) RNA 1997 14 1 89 103 8990402 PMC1369465

[B77] Schönhuber W Le Bourhis G Tremblay J Amann R Kulakauskas S Utilization of tmRNA sequences for bacterial identification BMC Microbiol 2001 14 1 20 10.1186/1471-2180-1-20 PMC55692 11560762

[B78] Barends S Kraal B van Wezel GP The tmRNA-tagging mechanism and the control of gene expression: a review Wires RNA 2011 14 2 233 246 10.1002/wrna.48 21957008

[B79] Barends S Zehl M Bialek S de Waal E Traag BA Willemse J Jensen ON Vijgenboom E van Wezel GP Transfer–messenger RNA controls the translation of cell-cycle and stress proteins in Streptomyces EMBO Rep 2009 14 2 119 125 20019758 10.1038/embor.2009.255 PMC2828758

[B80] Andre G Even S Putzer H Burguiere P Croux C Danchin A Martin-Verstraete I Soutourina O S-box and T-box riboswitches and antisense RNA control a sulfur metabolic operon of Clostridium acetobutylicum Nucleic Acids Res 2008 14 18 5955 5969 10.1093/nar/gkn601 18812398 PMC2566862

[B81] Cornillot E Nair RV Papoutsakis ET Soucaille P The genes for butanol and acetone formation in Clostridium acetobutylicum ATCC 824 reside on a large plasmid whose loss leads to degeneration of the strain J Bacteriol 1997 14 17 5442 5447 9286999 10.1128/jb.179.17.5442-5447.1997 PMC179415

[B82] Nair RV Bennett GN Papoutsakis ET Molecular characterization of an aldehyde/alcohol dehydrogenase gene from Clostridium acetobutylicum ATCC 824 J Bacteriol 1994 14 3 871 885 8300540 10.1128/jb.176.3.871-885.1994 PMC205125

[B83] Harris LM Welker NE Papoutsakis ET Northern, morphological, and fermentation analysis of spo0A inactivation and overexpression in Clostridium acetobutylicum ATCC 824 J Bacteriol 2002 14 13 3586 3597 10.1128/JB.184.13.3586-3597.2002 12057953 PMC135115

[B84] Fischer RJ Helms J Durre P Cloning, sequencing, and molecular analysis of the Sol operon of Clostridium acetobutylicum, a chromosomal locus involved in solventogenesis J Bacteriol 1993 14 21 6959 6969 8226639 10.1128/jb.175.21.6959-6969.1993 PMC206823

[B85] Nair RV Green EM Watson DE Bennett GN Papoutsakis ET Regulation of the sol locus genes for butanol and acetone formation in Clostridium acetobutylicum ATCC 824 by a putative transcriptional repressor J Bacteriol 1999 14 1 319 330 9864345 10.1128/jb.181.1.319-330.1999 PMC103564

[B86] Harris LM Blank L Desai RP Welker NE Papoutsakis ET Fermentation characterization and flux analysis of recombinant strains of Clostridium acetobutylicum with an inactivated solR gene J Ind Microbiol Biotechnol 2001 14 5 322 328 10.1038/sj.jim.7000191 11781808

[B87] Schiel B Nold N Dürre P Identification of a small noncoding RNA in Clostridium acetobutylicum. Programme for the 3rd Joint Conference of the German Society for Hygiene and Microbiology (Jahrestagung der Deutschen Gesellschaft für Hygiene und Mikorobiologie [DGHM]) and the Association for General and Applied Microbiology (Jahrestagung der Vereinigung für Allgemeine und Angewandte Mikrobiologie [VAAM]), 28 to 31 March 2010 2010 Hannover, Germany: GRV09:135

[B88] Trapnell C Pachter L Salzberg SL TopHat: discovering splice junctions with RNA-Seq Bioinformatics 2009 14 9 1105 1111 10.1093/bioinformatics/btp120 19289445 PMC2672628

[B89] Anders S Huber W Differential expression analysis for sequence count data Genome Biol 2010 14 10 R106 10.1186/gb-2010-11-10-r106 20979621 PMC3218662

[B90] Macke TJ Ecker DJ Gutell RR Gautheret D Case DA Sampath R RNAMotif, an RNA secondary structure definition and search algorithm Nucleic Acids Res 2001 14 22 4724 4735 10.1093/nar/29.22.4724 11713323 PMC92549

[B91] Lambert A Fontaine JF Legendre M Leclerc F Permal E Major F Putzer H Delfour O Michot B Gautheret D The ERPIN server: an interface to profile-based RNA motif identification Nucleic Acids Res 2004 14 W160 W165 10.1093/nar/gkh418 15215371 PMC441556

[B92] Gruber AR Lorenz R Bernhart SH Neuboock R Hofacker IL The Vienna RNA Websuite Nucleic Acids Res 2008 14 W70 W74 10.1093/nar/gkn188 18424795 PMC2447809

[B93] Hofacker IL Vienna RNA secondary structure server Nucleic Acids Res 2003 14 13 3429 3431 10.1093/nar/gkg599 12824340 PMC169005

