# Supplementary figures and images for: The Clostridium small RNome that responds to stress: the paradigm and importance of toxic metabolite stress in C. acetobutylicum
Source: BMC Genomics. 2013 Dec 4;14:849. doi: 10.1186/1471-2164-14-849 (PMC3879012; doi:10.1186/1471-2164-14-849)

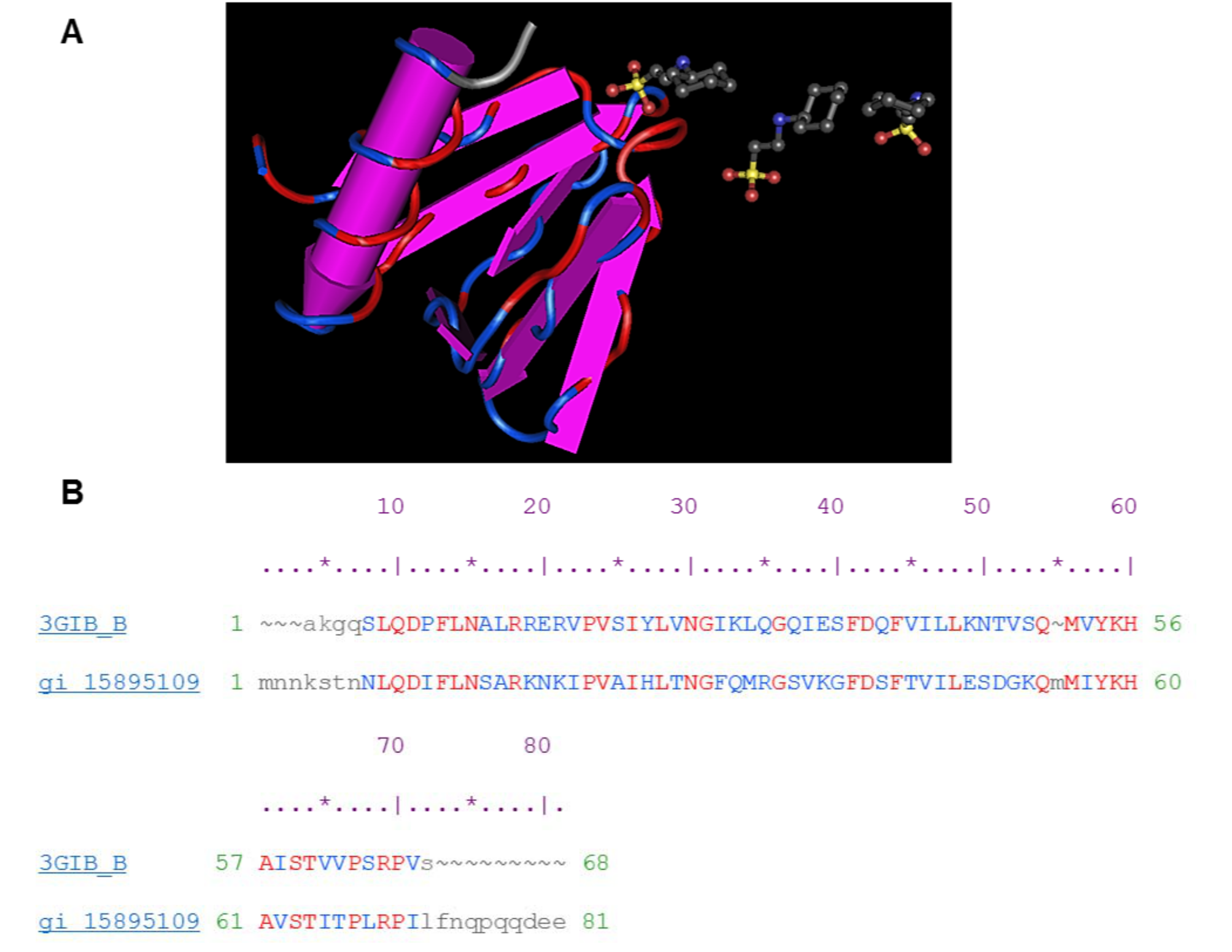

Supplement: Additional file 3: Figure S1 — CBLAST of the C. acetobutylicum hfq (CAC1834, gi_15895109) with the Hfq from E. coli (3GIB_B), reveals conservation in the secondary structure on the Hfq monomeric unit. (A) The α-β1-5 structural unit can be found to be conserved. (B) The corresponding conservation in the protein sequence is displayed below. [file 1471-2164-14-849-S3.tiff]

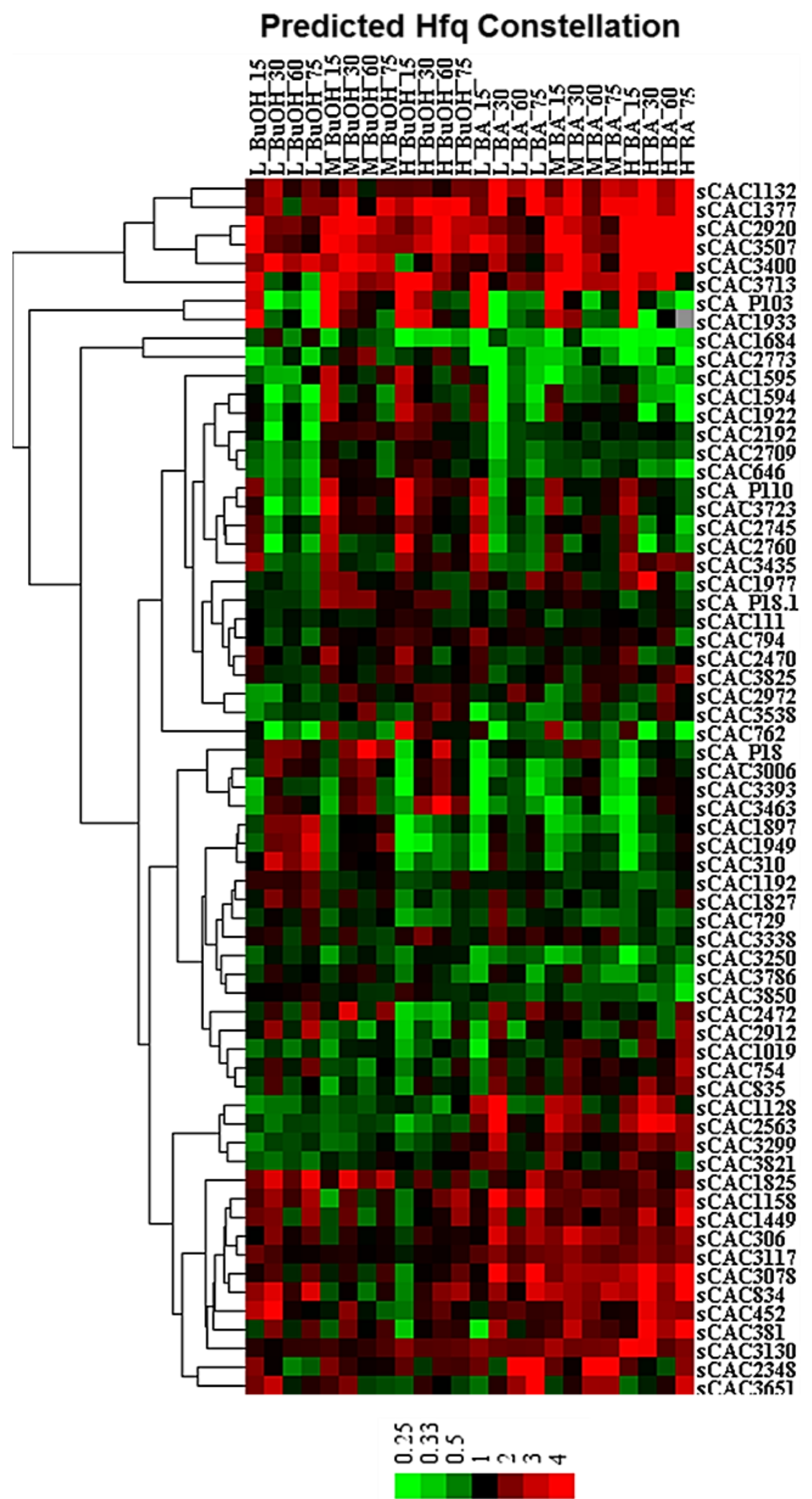

Supplement: Additional file 5: Figure S2 — Hierarchical clustering of the 65 sRNAs belonging to the Hfq constellation (see text for details). [file 1471-2164-14-849-S5.tiff]

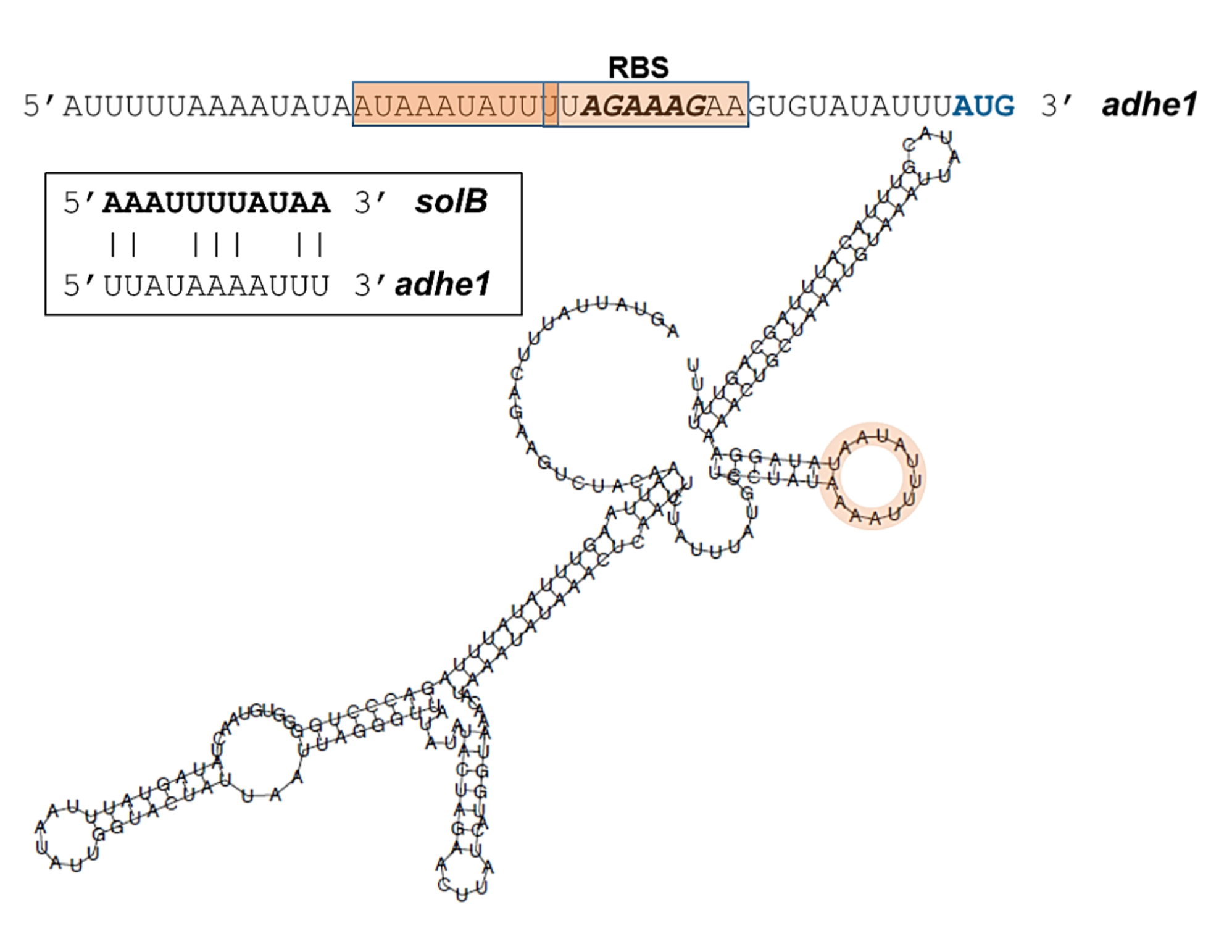

Supplement: Additional file 6: Figure S3 — Putative antisense binding of solB to the sol (adhE1-ctfA-ctfB) operon. [file 1471-2164-14-849-S6.tiff]

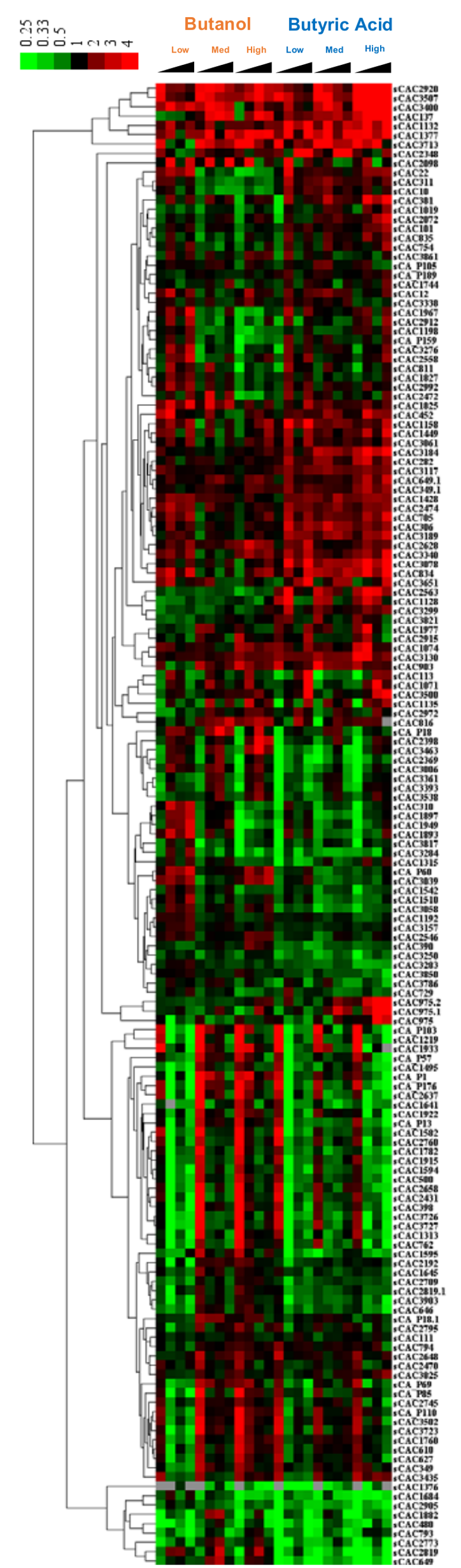

Supplement: Additional file 7: Figure S4 — Hierarchical clustering of 159 sRNAs under both butanol and butyric acid stress. [file 1471-2164-14-849-S7.tiff]
