# Supplementary material for: The Clostridium small RNome that responds to stress: the paradigm and importance of toxic metabolite stress in C. acetobutylicum
Source: BMC Genomics. 2013 Dec 4;14:849. doi: 10.1186/1471-2164-14-849 (PMC3879012; doi:10.1186/1471-2164-14-849)
Supplement: Additional file 2: Table S1 — Differential expression of annotated mRNAs of C. acetobutylicum. Pair-wise and point by point by comparison of each stress level to the no stress control using DEseq at a p-value ≤ 0.05. Table S2: Probes sequences used for Northern analysis. [file 1471-2164-14-849-S2.docx]

**SUPPLEMENTAL TABLES**

**Supplemental Table 1.**  Differential expression of annotated mRNAs of *C. acetobutylicum*. Pair-wise and point by point by comparison of each stress level to the no stress control using DEseq at an FDR of 0.05.

| **Butanol Stress Level** | **Upregulated** | **Downregulated** | **Butyrate Stress Level** | **Upregulated** | **Downregulated** |
| --- | --- | --- | --- | --- | --- |
| **Low (30 mM)** | 523 | 974 | **Low (30 mM)** | 156 | 262 |
| **Medium (60 mM)** | 107 | 105 | **Medium (40 mM)** | 133 | 320 |
| **High (90 mM)** | 264 | 671 | **High (50 mM)** | 299 | 285 |

**Supplemental Table 2.** Probes sequences used for Northern analysis.

| **sRNA** | **Probe Sequence** |
| --- | --- |
| tmRNA - sCAC834 | TTCCCTCCATGAAACGCCTACAAACA |
| 6S RNA - sCAC1377 | TACCGCTGCTCTTTCATTGACACC |
| SAM riboswitch - sCAC1132 | CTCATCTCTCAGCTAGCGCTGCAGG |
| sCAC3276 - INTEROP1858 | AGAGGCAAAGTAATAGCTTACCTCTAC |
| sCAC3463 - INTEROP1958 | AATATAATGGCTACGCCCAAACCCCGG |
| sCAC22 - INTEROP0009 | GGGACGAAAGACCGCGTTACCACCC |
| sCAC381 - INTEROP0218 | GCAAAAGCTGATACAAATACTATTGCGC |

**Reference**

1. Schiel B, N. Nold., Dürre P (2010) Identification of a small noncoding RNA in *Clostridium acetobutylicum*, abstr. GRV09, p. 135. Programme for the 3rd Joint Conference of the German Society for Hygiene and Microbiology (Jahrestagung der Deutschen Gesellschaft für Hygiene und Mikorobiologie [DGHM]) and the Association for General and Applied Microbiology (Jahrestagung der Vereinigung für Allgemeine und Angewandte Mikrobiologie [VAAM]), 28 to 31 March 2010, Hannover, Germany.
